# Toward Standardized Methodologies for Drug-Induced Proarrhythmia Classification: An In Silico Proof of Concept

**DOI:** 10.34133/csbj.0041

**Published:** 2026-04-10

**Authors:** Matteo Costi, Jose M. Ferrero, Jose F. Rodriguez Matas

**Affiliations:** ^1^ LaBS - In Silico Medicine Laboratory, Department of Chemistry, Materials and Chemical Engineering “G. Natta”, Politecnico di Milano, 20133 Milano, Italy.; ^2^ Center for Research and Innovation in Bioengineering (Ci2B), Universitat Politécnica de Valéncia, 46022 Valencia, Spain.

## Abstract

**Background:** The Comprehensive in vitro Proarrhythmia Assay integrates experimental electrophysiology with in silico simulations to improve the prediction of drug-induced proarrhythmic risk and to address regulatory limitations associated with traditional QT interval-based safety assessment. In recent years, increasing attention has been directed toward the arrhythmogenic potential of drugs, leading to the development of multiple computational frameworks. However, substantial methodological variability still exists across studies, including differences in electrophysiological models, biomarker selection, and population sampling strategies. Using a model of electrotonically coupled ventricular cardiomyocytes, this study proposes a proof of concept for a standardized in silico framework aimed at predicting drug-induced arrhythmic risk. **Methods:** A virtual population of ventricular cellular models was generated and calibrated using patient-derived electrophysiological data. Ten drugs representing 3 levels of proarrhythmic risk were evaluated both in isolated-cell simulations and in electrotonically coupled cellular networks. Drug classification was performed using a novel arrhythmic risk score, which integrates 8 established electrophysiological biomarkers. The analysis also evaluated the effects of electrotonic coupling, biomarker reduction, and cellular subsampling on the stability of the risk estimation. **Results:** The electrotonically coupled cell model reproduced the classifications obtained with the well-established isolated-cell model, demonstrating comparable predictive performance. However, reducing the number of biomarkers from 8 to 2 led to a substantial increase in false-negative classifications. Similarly, excessive reduction of the cellular population produced a non-negligible increase in the variability of the estimated risk score. **Conclusion:** Electrotonically coupled cellular networks provide a physiologically consistent framework that preserves the risk classifications obtained in isolated-cell simulations. The results indicate that reliable computational assessment of drug-induced proarrhythmic risk requires careful consideration of 3 key methodological elements: the electrophysiological model, the size of the simulated cellular population, and the number of biomarkers used for risk estimation.

## Introduction

The number of arrhythmia cases continues to increase worldwide [1], and cardiac ablation procedures are often not definitive treatments, with both short-term and long-term recurrence requiring redo interventions [2,3]. At the same time, the development and market approval of new therapeutic drugs is a lengthy, costly, and complex process, further constrained by strict regulatory requirements for commercialization [4].

Historically, the assessment of drug-induced proarrhythmic risk focused primarily on the evaluation of in vivo QT interval prolongation and blockade of the hERG potassium channel, which were considered surrogate markers for the risk of inducing life-threatening ventricular arrhythmias such as Torsades de Pointes (TdP) [5–7]. However, this paradigm proved overly restrictive. Clinical evidence has shown that certain drugs, such as amiodarone, which is widely used for the treatment of cardiac arrhythmias [8], can markedly prolong the QTc interval (even exceeding 550 ms) while rarely inducing TdP [5].

The need for a more comprehensive evaluation framework led to the development of the Comprehensive in vitro Proarrhythmia Assay (CiPA). This initiative integrates experimental electrophysiological measurements with in silico cellular simulations based on multichannel electrophysiological models of the cardiac action potential. The goal is to reconstruct drug-induced electrophysiological alterations, identify potential proarrhythmic events, and quantify arrhythmogenic risk using biomarker-based scoring approaches [5,9]. The CiPA initiative remains a proposed regulatory framework that is currently under evaluation.

Numerous electrophysiological models have been developed to reproduce ventricular action potentials and investigate proarrhythmic mechanisms, and many studies have proposed different biomarkers for identifying proarrhythmic events [10–13]. These models have been used to generate virtual populations of cellular models by varying the maximal conductances of key ionic currents [12,14,15], enabling the assessment of pharmacological cardiotoxic risk within the CiPA framework [14,15]. In several of these studies, abnormal repolarization events such as early afterdepolarizations have been used as the primary indicator of proarrhythmic behavior. As a result, substantial variability remains in the selection of biomarkers and methodological approaches used to assess pharmacological cardiotoxic risk.

In addition, most studies investigating drug-induced cardiotoxic behavior rely on isolated-cell models (ICMs) [14–16]. However, to achieve a more physiologically realistic representation of cardiac electrophysiology, it is necessary to account for electrotonic coupling between cardiomyocytes within the functional myocardial syncytium. Unlike isolated-cell simulations, in which cardiomyocytes behave as electrically independent units, simulations of coupled cellular networks allow cells to interact electrically and influence one another through gap-junction-mediated current flow.

Previous studies have explored proarrhythmic mechanisms using simplified tissue geometries. For example, Margara et al. [17] performed 3-dimensional simulations on a cuboid domain composed exclusively of endocardial cells with fibers aligned along the *z*-axis, preserving electrical and mechanical anisotropy while avoiding the computational burden of anatomically realistic ventricular geometries. Similarly, Yang et al. [18] extended ion-channel models to tissue-level simulations by generating pseudo-ECGs on idealized one-dimensional strands or small cellular networks, focusing on electrical activity and repolarization dynamics without reconstructing the full ventricular anatomy. In addition, the MRI-based 3-dimensional model proposed by Moreno et al. [19] incorporates electrotonic coupling through the monodomain equation, with myocardial conductivity tensors defining fiber-direction anisotropy and modulating electrical interactions throughout the virtual ventricle. However, none of these models has been explicitly used to perform drug-induced proarrhythmic risk assessment.

The present study aims to address this gap by performing a multiparametric analysis to identify the most relevant electrophysiological biomarkers for characterizing drug-induced proarrhythmicity. The resulting framework proposes a methodology that integrates multiple electrophysiological descriptors to provide a more robust and standardized assessment of cardiotoxic risk while reducing methodological variability across studies.

The proposed approach employs a simplified 3-dimensional cardiac tissue geometry in order to account for physiological electrotonic coupling between cells. The cellular electrophysiology is modeled using the human ventricular action potential model proposed by O’Hara et al. [20], combined with a population-of-models approach to capture variability in electrophysiological responses associated with drug-induced proarrhythmic risk. The methodology is evaluated using a set of 10 drugs with different cardiotoxic classifications.

## Methods

Numerous mathematical models have been developed in the literature to reproduce the action potential of ventricular cardiomyocytes and to investigate their electrophysiological behavior [20–26].

Over the years, several models have been proposed to study electrophysiological phenomena ranging from reentry mechanisms, such as rotors, to the prediction of pharmacological effects induced by newly administered drugs, such as the O’Hara–Rudy model [20]. In the present study, we adopt the O’Hara–Rudy monodomain formulation because of its validated electrophysiological fidelity and moderate computational cost.

### Mathematical methods

#### Isolated-cell model

The O’Hara–Rudy model is widely used in the scientific literature to characterize the human ventricular action potential and to investigate morphological alterations following drug administration, while also accounting for calcium handling and calcium buffering mechanisms. It was selected by a consensus of in silico modelers for use in the CiPA initiative [27,28] and serves as the baseline model for the population of models adopted in this study.

The ICM is described as:

$$C_{m}\frac{{\mathrm{dV}}_{m}}{\mathrm{dt}}=-\left(I_{\mathrm{tot}}+I_{\text{stim}}\right)$$ (1)

where $V_{m}$ is the transmembrane potential, $C_{m}$ is the membrane capacitance, $I_{\text{stim}}$ represents the externally applied stimulus current, and $I_{\mathrm{tot}}$ is the sum of all ionic membrane currents:

$$\begin{array}{c} \begin{array}{l} I_{\mathrm{tot}}=I_{\mathrm{Na}}+I_{\mathrm{NaL}}+I_{\mathrm{CaL}}+I_{\text{to}}+I_{K1}+I_{\mathrm{Kr}}+I_{\mathrm{Ks}}+I_{\text{NaCa}}\\ \;+I_{\mathrm{NaK}}+I_{\mathrm{Cap}}+I_{\mathrm{Nab}}+I_{\mathrm{Cab}}+I_{\mathrm{Kb}} \end{array} \end{array}$$ (2)

where $I_{\mathrm{Na}}$ is the fast sodium (${\mathrm{Na}}^{+}$) current; $I_{\mathrm{NaL}}$ is the late sodium current; $I_{\mathrm{CaL}}$ is the L-type calcium (${\mathrm{Ca}}^{2+}$) current; $I_{\text{to}}$ is the transient outward potassium ($K^{+}$) current; $I_{K1}$ is the inward rectifier potassium current; $I_{\mathrm{Kr}}$ is the rapid delayed rectifier potassium current; $I_{\mathrm{Ks}}$ is the slow delayed rectifier potassium current; $I_{\text{NaCa}}$ is the sodium-calcium exchanger current; $I_{\mathrm{NaK}}$ is the sodium–potassium pump current; $I_{\mathrm{Cap}}$ is the sarcolemmal calcium pump; and $I_{\mathrm{Nab}}$, $I_{\mathrm{Cab}}$, and $I_{\mathrm{Kb}}$ represent background currents.

#### Tissue model

Electrotonic coupling represents the ability of cardiomyocytes to electrically interact with each other, mutually influencing their electrophysiological behavior. Since cardiomyocytes operate within the functional syncytium of the heart, it is necessary to introduce an electrotonically coupled cell model (ECCM) capable of reproducing this physiological environment in order to investigate cellular behavior following drug administration.

In this study, the propagation of the electrical signal within the tissue was simulated using the monodomain model

$$\begin{array}{c} \nabla \left(D\nabla V\right)=\left(C_{m}\frac{\partial V_{m}}{\partial t}+I_{\mathrm{tot}}+I_{\text{stim}}\right)\;\text{in}\;\Omega \end{array}$$ (3)

$$n\left(D\nabla V\right)=0\;\text{in}\;\partial \Omega$$ (4)

where D is the effective conductivity tensor, Ω and ∂Ω represent the computational domain and its boundary, respectively, and n denotes the outward normal vector to ∂Ω. Equations (3) and (4) replace Eq. (1) when electrotonic coupling is considered.

#### Drug models

Drugs act at the ionic channel level by modifying the maximum conductance of specific channels. To simulate these pharmacological effects, the pore block model is commonly used, as it provides a quantitative description of ionic channel inhibition through a block coefficient $B_{k}$ defined as:

$$B_{k}=\frac{1}{1+{\left(\frac{\left[C\right]}{{\mathrm{IC}}_{50}}\right)}^{n}}$$ (5)

where $\left[C\right]$ is the plasma concentration of the compound, *n* is the Hill coefficient, and ${\mathrm{IC}}_{50}$ is the half-maximal inhibitory concentration [29]. Table 1 reports the values of ${\mathrm{IC}}_{50}$, the Hill coefficient *n*, and the effective free therapeutic plasma concentration (EFTPC) for the 10 analyzed compounds. The experimental values of IC_50_ and Hill coefficients were obtained from Ref. [30].

**Table 1.** Parameters of the pore block model for the different compounds, IC_50_, in μM, and Hill coefficient, *n*, in parenthesis, together with the EFTPC in μM

Drugs	$I_{\mathrm{Na}}$	$I_{\mathrm{CaL}}$	$I_{\text{to}}$	$I_{\mathrm{Kr}}$	$I_{\mathrm{Ks}}$	$I_{K1}$	$I_{\text{NaCa}}$	$I_{\mathrm{NaK}}$	EFTPC
Amiodarone	4.577	1.281	3.758	0.941	13.390	∞	∞	∞	0.778
	(0.7)	(0.6)	(0.4)	(0.6)	(1)	(1)	(1)	(1)	
Bepridil	2.929	2.806	∞	0.149	∞	∞	∞	∞	0.0315
	(1.2)	(0.6)	(1)	(0.9)	(1)	(1)	(1)	(1)	
Diltiazem	∞	0.112	∞	6.569	∞	∞	∞	∞	0.1275
	(1)	(0.7)	(1)	(0.8)	(1)	(1)	(1)	(1)	
Dofetilide	∞	∞	0.018	0.001	∞	∞	∞	∞	0.0021
	(1)	(1)	(1)	(0.6)	(1)	(1)	(1)	(1)	
Flecainide	6.677	25.599	9.266	0.692	∞	∞	∞	∞	0.7529
	(1.9)	(1.4)	(0.7)	(0.8)	(1)	(1)	(1)	(1)	
Mibefradil	5.866	0.652	∞	0.307	∞	33.802	∞	∞	0.0106
	(1)	(1.1)	(1)	(0.9)	(1)	(1)	(1)	(1)	
Moxifloxacin	922.727	∞	∞	93.041	50.321	∞	∞	∞	3.5625
	(1)	(1)	(1)	(0.6)	(1)	(1)	(1)	(1)	
Quinidine	18.815	∞	3.847	0.343	4.899	∞	∞	∞	0.8429
	(1)	(1)	(1.3)	(1)	(1.4)	(1)	(1)	(1)	
Sotalol	∞	5,976.923	∞	86.369	4,762.745	3,340.415	∞	∞	14.6864
	(1)	(1)	(1)	(0.9)	(1)	(1)	(1)	(1)	
Verapamil	∞	0.202	∞	0.499	∞	∞	∞	∞	0.045
	(1)	(1.1)	(1)	(1.1)	(1)	(1)	(1)	(1)	

Recent approaches have extended the pore block formulation by incorporating dynamic drug–channel interactions. For instance, Markov model-based approaches explicitly simulate state-dependent binding to open, inactivated, and closed channel states, as well as trapping dynamics, providing more accurate predictions of Hill curves and action potential prolongation compared with static models based solely on IC_50_ values [31].

### Modeling variability and electrically coupled model

A crucial aspect in assessing drug-induced proarrhythmic risk and understanding the underlying mechanisms that contribute to drug-induced arrhythmias is the incorporation of electrophysiological variability. Previous studies [12,27,32,33] have demonstrated the importance of intrinsic cellular heterogeneity in improving the accuracy and predictive capability of computational simulations related to electrophysiology and drug risk assessment.

To characterize physiological variability in the ionic currents described in Eq. (2), conductances were sampled within the range of −80% to +200% of their reference values using Latin hypercube sampling, which ensures a uniform and balanced exploration of the entire parameter space [12]. This procedure generated approximately 100,000 highly heterogeneous action potentials, which were subsequently filtered according to exclusion criteria related to alternans, repolarization abnormalities, and self-stimulation, resulting in a set of approximately 80,000 stable and physiologically plausible models [9].

Calibration against experimental data was performed using percentiles of 5 action potential biomarkers (APD20, APD50, APD90, amplitude, and resting membrane potential). Data are reported in Table 2. This approach was preferred over the use of mean and standard deviation in order to preserve the empirically observed non-Gaussian distributions.

**Table 2.** Calibration percentiles (5° to 95°) related to ventricular action potentials’ biomarkers

Biomarker	$5^{\circ }$ pct.	$95^{\circ }$ pct.
RMP (mV)	−95	−85
APA (mV)	102.7	116.4
APD_20_ (ms)	138	188
APD_50_ (ms)	181	251
APD_90_ (ms)	250	331

Finally, to investigate the effect of electrotonic coupling in a tissue context, a cuboid measuring 1.8×1.8×18 mm^3^ with fibers aligned along the longitudinal (*z*) axis was constructed (details and associated data are provided in the Supplementary Materials). Each node of the parallelepiped was assigned a distinct action potential model, resulting in a computational domain composed of 2,160 trilinear hexahedral elements and 2,989 nodes, each associated with a different action potential model.

Simulations were carried out using both an ICM and a tissue model incorporating electrotonic coupling between cells. This approach allowed cardiomyocytes to interact with each other and behave as they would within the cardiac functional syncytium. The results obtained from the ICM and the ECCM were compared in order to analyze their respective behaviors following drug administration. To ensure a consistent comparison, the same initial drug-free cellular population was used for both the ICM and the ECCM. This approach allowed for the assessment of the effects of drug administration at both cellular and tissue levels.

The model parameters were set as follows: longitudinal conductance $\sigma_{L}=0.0023\;S/\mathrm{cm}$, transverse-to-longitudinal conductance ratio $r=\sigma_{T}/\sigma_{L}=0.35$, and membrane capacitance $C_{m}=1.0\;\mu F/{\mathrm{cm}}^{2}$.

The cuboid was treated as a transversely isotropic medium, with the conductivity tensor defined as:

$$D=\sigma_{L}\left[\left(1-r\right)\;n⊗n+rI\right]$$ (6)

where **n** denotes the local fiber direction and **I** is the identity tensor.

Monodomain simulations were performed using an implicit solver with a time step of 0.02 ms, providing a balance between numerical accuracy and computational efficiency.

### Pharmacological modeling

We selected a set of 10 pharmacological compounds in order to include:

1. Proarrhythmic drugs: amiodarone, bepridil, dofetilide, flecainide, moxifloxacin, and quinidine.

2. Non-proarrhythmic drugs: diltiazem, mibefradil, and verapamil.

3. Borderline drugs: sotalol.

This preliminary risk categorization is based on a previous study by Zhou et al. [15], which characterizes the risk of inducing TdP associated with drug administration. In this study, the term “borderline drugs” is used to refer to compounds associated with a conditional TdP risk score.

During the development process of new pharmacological compounds, rigorous analyses are conducted prior to market release. To determine the non-proarrhythmic nature of new drugs, it is common practice to test compounds at different concentrations in order to identify potential proarrhythmic behaviors [14,16,34,35]. In this study, all drugs were tested at 5 different concentrations, each expressed as a multiple of the EFTPC, specifically at 1×, 3×, 10×, 30×, and 100× the EFTPC.

High drug concentrations were also investigated to simulate the effects of impaired renal function, as observed in kidney disease, where metabolic dysfunction can lead to excessive drug accumulation [36].

### Multiparametric analysis for identifying critical biomarkers

In the literature, numerous studies propose different biomarkers as classifiers for TdP risk [12,16,34,35]. However, there is currently no standardized or universally accepted methodology for comprehensively assessing drug-induced proarrhythmic risk, in terms of either biomarker selection or threshold definition.

Using the CiPAORdv1.0 model, the Food and Drug Administration (FDA) has recently shifted its approach from the analysis of action potential morphology to the evaluation of the overall electrical charge balance during action potential expression (qNET/TMS) [37]. Nevertheless, several European regulatory agencies, including the European Medicines Agency, continue to rely on QT interval prolongation as a primary metric to assess the proarrhythmic potential of drugs, an approach also adopted by resources such as CredibleMeds [38].

Several studies have employed different parameters, including action potential duration (APD) at specific percentages of repolarization, the maximum derivative during the resting phase, early afterdepolarizations, triangulation, abnormal peaks, intracellular calcium concentrations, and other electrophysiological indicators [10,11,16,35,39].

In the present study, rather than relying on individual biomarkers to assess the proarrhythmic potential of drugs, we integrated several of the most relevant biomarkers and evaluated their combined effectiveness. This approach aims to identify the most appropriate indicators while minimizing the risk of underestimating proarrhythmic behavior.

All biomarkers used in our analysis were selected to capture a heterogeneous range of instabilities in action potential morphology. For this reason, we adopted the term “arrhythmic risk score” (ARS) instead of “TdP risk score”. This choice provides a broader perspective on arrhythmia risk that extends beyond TdP alone.

These biomarkers were implemented to detect variations indicative of potential arrhythmic events, ensuring accuracy in the overall assessment. We developed an algorithm that filters action potentials exhibiting instability characteristics. Each time a biomarker detects a proarrhythmic event, it contributes to the computation of the probability that a specific arrhythmic event occurs during drug administration. This framework therefore allows both a comprehensive evaluation of action potential instabilities and an estimation of the likelihood of arrhythmic events in response to pharmacological intervention.

**Fig. 1.**
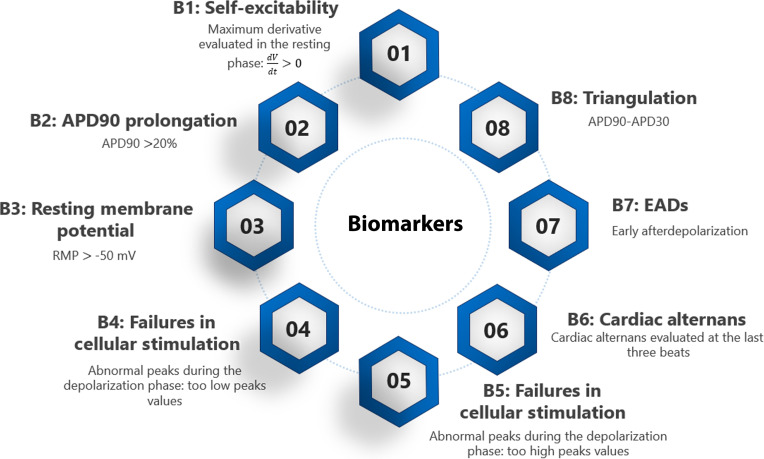
The 8 biomarkers used in the analysis are illustrated. Each biomarker detects a specific proarrhythmic event and marks the corresponding action potential as unstable.

Figure 1 shows a schematic representation of the 8 biomarkers adopted to identify instabilities in action potential morphology.

B1 represents the maximum derivative evaluated during the resting phase, calculated using a threshold value of 0.01.

B2 is associated with APD90 prolongation. APD90 was first calculated for the drug-free population and then for the same population after drug administration. B2 was subsequently calculated as the percentage increase in APD90 in the drug-treated population relative to the drug-free population. A proarrhythmic event was classified as such when this percentage increase exceeded 20%.

B3 is associated with the resting membrane potential, with a proarrhythmic event identified when this biomarker exceeds the threshold of −50 mV.

B4 and B5 are biomarkers related to abnormalities in cellular stimulation, reflecting nonphysiological values of the action potential peak. To better characterize these events, low peaks (below 0 mV) and high peaks (above 50 mV) were analyzed separately, corresponding to understimulation and overstimulation, respectively.

B6 is associated with cardiac alternans, evaluated over the last 3 beats. Cardiac alternans was considered valuable when the APD90 of 1 of the last 3 beats differed by more than 5% from the other 2.

B7 characterizes early afterdepolarizations observed in the last beat.

B8 represents triangulation, calculated as the difference between APD90 and APD30.

Additional details related to the implementation and thresholds are reported in the Supplementary Materials

### Risk score index and proarrhythmicity thresholds

The risk index is defined as a composite measure, hereafter designated as ARS, since the biomarkers employed are not specific to TdP alone but also characterize other ventricular arrhythmias, i.e., cardiac alternans, which are known to be precursors of ventricular tachycardia such as bigeminal premature ventricular contractions or indicators of supraventricular tachycardia [40]. The following formula for ARS index, $x_{i}$, has been computed both for the ICM and for ECCM:

$$x_{i}=\sum_{j=1}^{8}w_{j}p_{i,j}$$ (7)

where *i* is the index of the 10 drugs and *j* cycles for the 8 biomarkers. For each biomarker *j*, the corresponding weight is obtained by:

$$w_{j}=\frac{\mathrm{AUC}}{\sum_{m=1}^{8}{\mathrm{AUC}}_{m}}$$ (8)

computing the AUC of binary ROC curve “Pro vs. Non Pro” over all the 10 drugs. Thus, the risk index is a weighted sum of single probabilities bounded to each biomarker. As a consequence, the probability $p_{i,j}$ is defined as:

$$p_{i,j}=\frac{\sum_{k=1}^{5}M_{j,k}^{i}/C_{k}}{N\;\sum_{k=1}^{5}1/C_{k}}$$ (9)

where *N* is the total number of sampling points, i.e., total number of cells in the population. In this context, *k* iterates over the 5 tested concentrations (1×, 3×, 10×, 30×, and 100× EFPTC), and $M^{i}$ is an 8 × 5 matrix whose entries $M_{j,k}^{i}$ denote the number of cells classified as proarrhythmic by biomarker *j* at concentration *k*, $C_{k}$, for each *i* drug. The factor $\frac{1}{C_{k}}$ is included to assign greater weight to lower concentrations. Hence, the matrix has 8 rows (one per biomarker) and 5 columns (one per drug concentration), and each element reflects the count of proarrhythmic events detected by a specific biomarker at a given concentration. Thus, global indices for each compound are obtained. Upon computing the risk scores, $x_{i}$, classification labels are then assigned: 0 for low risk, 1 for borderline, and 2 for high risk.

Thus, we introduce 2 thresholds *l* and *h* with l<h, to define the classification rule:

$${\hat{y}}_{i}\left(\ell ,\;h\right)=\left\{\begin{array}{cc} 0, & x_{i}\leq \ell \\ 1,\; & \ell <x_{i}\leq h\\ 2, & \;x_{i}>h \end{array}\right.$$ (10)

For each class $\mathrm{cl}=\left\{0,1,2\right\}$ in the classification problem, we define:

• **True positives** (${\mathrm{TP}}_{\mathrm{cl}}$): the number of samples that truly belong to class $y_{i}=\mathrm{cl}$ and that are classified as ${\hat{y}}_{i}=\mathrm{cl}$.
• **False positives** (${\mathrm{FP}}_{\mathrm{cl}}$): the number of samples that do not belong to class $y_{i}\neq \mathrm{cl}$ but are classified as ${\hat{y}}_{i}=\mathrm{cl}$.
• **False negatives** (${\mathrm{FN}}_{\mathrm{cl}}$): the number of samples that truly belong to class $y_{i}=\mathrm{cl}$ but are classified in a different class ${\hat{y}}_{i}\neq \mathrm{cl}$.

Note that ${\mathrm{TP}}_{\mathrm{cl}}$, ${\mathrm{FP}}_{\mathrm{cl}}$, and ${\mathrm{FN}}_{\mathrm{cl}}$ are extracted from the confusion matrix generated upon the classification process.

### Computation of the thresholds *l* and *h*

The performance of the classification algorithm depends on the value of the thresholds *l* and *h* defining the 3 classes. The optimal selection of these thresholds is performed as follows. Once ${\mathrm{TP}}_{\mathrm{cl}}$, ${\mathrm{FP}}_{\mathrm{cl}}$, and ${\mathrm{FN}}_{\mathrm{cl}}$ are computed, we define ${\text{Precision}}_{\mathrm{cl}}$ and ${\text{Recall}}_{\mathrm{cl}}$ as:

$$\begin{array}{c} {\text{Precision}}_{\mathrm{cl}}=\frac{{\mathrm{TP}}_{\mathrm{cl}}}{{\mathrm{TP}}_{\mathrm{cl}}+{\mathrm{FP}}_{\mathrm{cl}}},\;{\text{Recall}}_{\mathrm{cl}}=\frac{{\mathrm{TP}}_{\mathrm{cl}}}{{\mathrm{TP}}_{\mathrm{cl}}+{\mathrm{FN}}_{\mathrm{cl}}} \end{array}$$ (11)

where ${\text{Precision}}_{\mathrm{cl}}$ and ${\text{Recall}}_{\mathrm{cl}}$ respectively quantify the reliability of predictions for class cl and the proportion of true cl instances that are correctly identified. Thus, we define the performance metric for class cl, $F1_{\mathrm{cl}}$, as the harmonic mean of ${\text{Precision}}_{\mathrm{cl}}$ and ${\text{Recall}}_{\mathrm{cl}}$

$$F1_{\mathrm{cl}}=\frac{2\;{\text{Precision}}_{\mathrm{cl}}\;{\text{Recall}}_{\mathrm{cl}}}{{\text{Precision}}_{\mathrm{cl}}+{\text{Recall}}_{\mathrm{cl}}}$$ (12)

Since we have more classes, the ${\text{Macro}}_{F1}$ is defined as the mean of the 3 $F1_{\mathrm{cl}}$:

$${\text{Macro}}_{F1}=\frac{1}{3}\sum_{\mathrm{cl}\;\in \;\left\{0,1,2\right\}}F1_{\mathrm{cl}}$$ (13)

To optimize the low threshold *l* and high threshold *h* for maximum ${\text{Macro}}_{F1}$, we first compute the continuous risk score $x_{i}$ for each sample and then run a 2-dimensional grid search. We start by identifying 3 key statistics of the *x* distribution: its minimum value, its median, and its maximum. Next, we construct 2 vectors of 40 equally spaced candidate thresholds: one for *l*, spanning from the minimum up to the median, and one for *h*, spanning from the median up to the maximum. For each pair (*l*, *h*) with *l* < *h*, we discretize each $x_{i}$ into one of 3 classes $\mathrm{cl}=\left\{0,1,2\right\}$, compute the confusion matrix, and calculate ${\text{Precision}}_{\mathrm{cl}}$, ${\text{Recall}}_{\mathrm{cl}}$ and $F1_{\mathrm{cl}}$ for each class, *cl*, and then ${\text{Macro}}_{F1}$ score. We choose the pair (l^*^, h^*^) that maximizes this score. Defining the search intervals this way, anchored at the minimum, median, and maximum, ensures an exhaustive yet efficient exploration of the relevant range (avoiding l>h combinations), offers robustness to outliers, and limits the computation to only tens of thousands of evaluations.

Hence, the analysis is structured in 2 phases: in the first, binary weights are computed to optimize the continuous index for identifying proarrhythmic drugs (the primary clinical objective); in the second, the controlled incorporation of the ${\text{Macro}}_{F1}$ score defines the “borderline” category while preserving methodological robustness and transparency. In imbalanced 3-class problems, overall accuracy often overlooks minority groups. By averaging the F1 score for each class equally, ${\text{Macro}}_{F1}$ ensures that the performance in the clinically critical “borderline” and “high” categories is as visible as in “low-risk” categories, leading to a more fair and more informative evaluation [41].

We assessed the robustness of the Macro-F1 score using leave-one-out cross-validation (LOOCV). In each iteration, one compound was held out while the remaining compounds were used as the training set. Within each fold, biomarker weights were recomputed from the ROC–AUC values calculated on the training drugs, and the classification thresholds were optimized on the same training set. Performance was then evaluated on the excluded compound. The final LOO − Macro-F1 score is the average of the Macro-F1 values across the 10 cross-validation folds. F1 LOO = 0.859; accuracy of LOO = 90%.

Permutation test (*B* = 1,000) has been applied, confirming that this performance is highly unlikely under label randomization (*P* ≈ 0.007). We finally estimated confidence intervals for thresholds applying another bootstrap, resampling the 10-drug set (*B* = 1,000) with replacement, re-estimating all (*l_j_*, *h_j_*).

#### Mathematical framework for subpopulations risk indexes

We follow the same approach as before, now generalizing the mathematical framework to accommodate subpopulations.

For each drug d=1,…,D, iteration t=1,…,T, biomarker j=1,…,J, and concentration c=1,…,C, define the indicator

$$X_{d,i,j}^{\left(t\right)}\left(c\right)\in \left\{0,\;1\right\},i=1,\ldots ,N,$$ (14)

which equals 1 if cell *i* in sample $\chi_{d}^{\left(t\right)}$ exhibits event *j* at concentration $C_{c}$, and 0 otherwise.

The number of observed events is

$$N_{d,t}\left(j,\;c\right)=\sum_{i=1}^{n}X_{d,i,j}^{\left(t\right)}\left(c\right)$$ (15)

The empirical instability probability for biomarker *j*, aggregated across concentrations, is

$${\hat{p}}_{d,t}^{\left(j\right)}=\sum_{c=1}^{C}w_{c}\;\frac{N_{d,t}\left(j,\;c\right)}{N},\;w_{c}=\frac{\omega \left(c\right)}{\sum_{c^{\prime }=1}^{C}\omega \left(c^{\prime }\right)}$$ (16)

where $\omega \left(c\right)\geq 0$ is an arbitrary weight, in this case set to $\omega \left(c\right)\;=\;1\;/\;c$.

The inverse-concentration weighting was introduced to emphasize drug effects occurring near clinically relevant exposure levels. While higher multiples of the EFTPC are commonly used in in silico and in vitro cardiotoxicity studies to explore extreme pharmacological scenarios, arrhythmogenic events occurring close to therapeutic concentrations are more informative for clinical risk assessment. Therefore, the weighting scheme progressively down-weights supratherapeutic concentrations while preserving their contribution to the overall instability profile.

Thus, eventually at each iteration *t* and for each drug *d*, we assign the score

$$r_{d,t}^{\left(J\right)}\;=\;\sum_{j=1}^{J}w_{j}\;{\hat{p}}_{d,t}^{\left(j\right)}$$ (17)

where *w_j_* are the AUC weights normalized. Repeating this for t=1,…,T yields the matrix

$$R\;=\;{\left[r_{d,t}\right]}_{d=1,\ldots ,D,\;t=1,\ldots ,T}$$ (18)

which represents the master matrix from which we derive all statistical parameters.

#### Mathematical framework for subsets of the full 8-biomarker set

To assess the impact of arbitrary biomarker selection and sample size, we partitioned the full 8-biomarker set into every possible 2-biomarker combination and repeated the entire analysis for each subset.

Setting *J* = 8, all possible unordered pairs are given by:

$$\begin{array}{c} P=\left\{\left(i,\;j\right)|1\leq i<j\leq 8\right\},\;|\;P\;|=28 \end{array}$$ (19)

For each pair $\left(i,\;j\right)\in P$ and for every drug d and iteration t, we have already computed risk index. For each pair, Eq. (16) becomes:

$$\begin{array}{c} r_{d}^{\left(i,j,t\right)}=\alpha P_{d,i}^{\left(t\right)}\;+\;\left(1-\alpha \right)P_{d,j}^{\left(t\right)},\left(\alpha =0.5\right) \end{array}$$ (20)

The value of α was fixed at 0.5 after computing the optimal α that minimizes the overall root mean square error (RMSE):

$$\begin{array}{c} \text{RMSE}\left(\alpha \right)=\sqrt{\frac{1}{DT}\sum_{d=1}^{D}\sum_{t=1}^{T}{\left(\alpha p_{d,t}^{\left(i\right)}+\left(1-\alpha \right)p_{d,t}^{\left(j\right)}-r_{d,t}^{\left(8\right)}\right)}^{2}} \end{array}$$ (21)

The comparison of the 2 α values was made as ΔRMSE=${\text{RMSE}}^{\left(0.5\right)}-{\text{RMSE}}^{\left(\alpha^{*}\right)}$.

The improvement at the optimized $\alpha^{*}$ = 0.75 is real but too small to justify its adoption. ΔRMSE is on the order of 0.02 to 0.03, which falls below the intrinsic uncertainty of the bootstrap. This marginal gain does not compensate for the added complexity, risks overfitting, and makes the choice of α = 0.5 preferable a priori by avoiding any possibility of cherry-picking.

It should be emphasized that, in this analysis, the 8-biomarker ARS is not intended to represent an external clinical ground truth. Rather, it serves as an internal reference model that aggregates the full set of electrophysiological descriptors considered in this study. Consequently, comparisons between 2-biomarker subsets and the full 8-biomarker score should be interpreted as a methodological assessment of information loss due to biomarker reduction, rather than as a direct evaluation of clinical predictive performance. In this framework, error metrics such as RMSE, bias, false positives, and false negatives quantify the deviation of reduced biomarker sets from the composite internal model.

Furthermore, we assessed the robustness of the Macro-F1 score using LOOCV. In each iteration, one compound was held out while the remaining compounds were used as the training set. Within each fold, biomarker weights were recomputed from the ROC–AUC values calculated on the training drugs, and the classification thresholds were optimized on the same training set. Performance was then evaluated on the excluded compound.

To conclude, despite the fact that Passini et al. [42] have demonstrated that smaller but well-designed populations can achieve results as accurate as those obtained with larger populations, such studies often rely on biomarkers related to mechanical contractility, such as diastolic calcium levels or the electromechanical window. In contrast, when focusing exclusively on electrophysiological biomarkers associated with action potential morphology, using sample sizes that are too small may compromise the accuracy of the final drug-induced proarrhythmic risk. This topic will be discussed in greater detail in the results and discussion sections.

### Statistical analysis

Interpreting results from computational simulations involving multiple samples can be challenging and occasionally misleading, highlighting the need for careful consideration of sample size. In particular, drawing reliable conclusions regarding mechanisms and effects in biomedical and pharmacological research requires continuous scrutiny due to the substantial variability associated with human physiology.

Therefore, establishing statistically robust outcomes that link hypotheses to results is essential and depends strongly on the selection of an appropriate sample size. In human tissue, a large number of cells coexist. In the myocardium, for instance, the number of cardiomyocytes has been estimated at approximately 3.2±0.75 billion [43]. Assuming a left ventricular volume ranging between 100 and 120 cm^3^ [44], and adopting a simplified approximation in which cardiomyocytes are uniformly distributed within the tissue, the number of cells contained in the tissue preparation used in this study can be estimated to be approximately 2 × 10^6^.

Simulating a single action potential on a workstation equipped with 32 GB of RAM and an Intel Core i7-10870H CPU requires approximately 30 s. Consequently, large-scale simulations involving more than $10^{6}$ cells, across 10 drugs and 5 concentration levels, become computationally prohibitive. While bidomain formulations or GPU-accelerated schemes can provide speedups ranging from 10× to 200× on specialized hardware, these solutions are often less accessible on standard HPC clusters [45].

To balance statistical power, reproducibility, and computational cost, we therefore adopted a cuboidal mesh composed of 2,160 elements, corresponding to 2,989 virtual ventricular cardiomyocytes modeled using the endocardial O’Hara–Rudy formulation. Recognizing that analyses based on small cellular cohorts may lead to substantial misclassification of drug proarrhythmic risk, we compared results obtained using both limited and larger sample sizes.

Drawing inspiration from the work of Zhou et al., who reported simulations based on 107 cells, we implemented a bootstrapping procedure starting from the full pool of 2,989 virtual cardiomyocytes.

We randomly extracted progressively smaller subsamples from the full population of simulated cells, including subsets comparable to those used in smaller cohorts reported in the literature (e.g., the 100-cell population considered by Zhou et al. [15]). For each subsample size, the extraction procedure was repeated 200 times (an iteration count determined through preliminary sensitivity testing) in order to construct empirical distributions of the resulting ΔARS values. Additional details are provided in the Supplementary Materials.

By comparing these distributions with the results obtained using the full cellular population, we show that stable and reliable risk classifications are achieved only when sufficiently large sample sizes and the complete set of 8 biomarkers are considered. This methodology aligns with uncertainty quantification practices reported in the literature and highlights the importance of adequately sized cellular cohorts for reliable proarrhythmic risk assessment [46,47].

### Visualization plots

The bubble plot relates the RMSE between the risk score computed using a pair of biomarkers and the reference ICM-8 score to the median bias between the 2 estimates. The horizontal axis represents the average deviation of the 2-biomarker pair from the absolute value of the reference risk score, while the vertical axis indicates whether the 2-biomarker model tends to underestimate the risk (negative bias, FN-driven) or overestimate it (positive bias, FP-driven) relative to ICM-8. Bubble size and color encode the magnitude of false negatives or false positives, allowing pairs that achieve both low overall error and a balanced distribution of type I and type II errors to be visually identified.

In contrast, the grouped bar plot explicitly displays, for each of the 28 biomarker pairs, the percentage of false negatives and false positives calculated across all single-cell observations. This representation makes it immediately clear which biomarker pairs maximize each type of error and by how much, without requiring interpretation based on position or color.

For the bubble plot, the procedure is as follows. For each biomarker pair, 200 subsets of 100 cells were generated. For each subset, the vector of mean risk scores per drug was computed and the overall RMSE relative to the ICM-8 score (i.e., the weighted combination of the 8 biomarkers) was evaluated. Subsequently, the median risk score for each drug was calculated under both the 8-biomarker model and the 2-biomarker pair, and their median bias was computed (${\text{median}}_{2}-{\text{median}}_{8}$). Finally, across all cells, the percentage of cases in which the pair predicted “proarrhythmic” when the reference model did not (false positives) or failed to predict it when the reference did (false negatives) was determined.

Bubble plots are divided into 2 panels: one corresponding to bias<0 (FN-driven) and the other to bias>0 (FP-driven). Bubble area is proportional to the total error (FP+FN), or a function of it, while color scales with the percentage of false negatives in the left panel and false positives in the right panel.

**Fig. 2.**
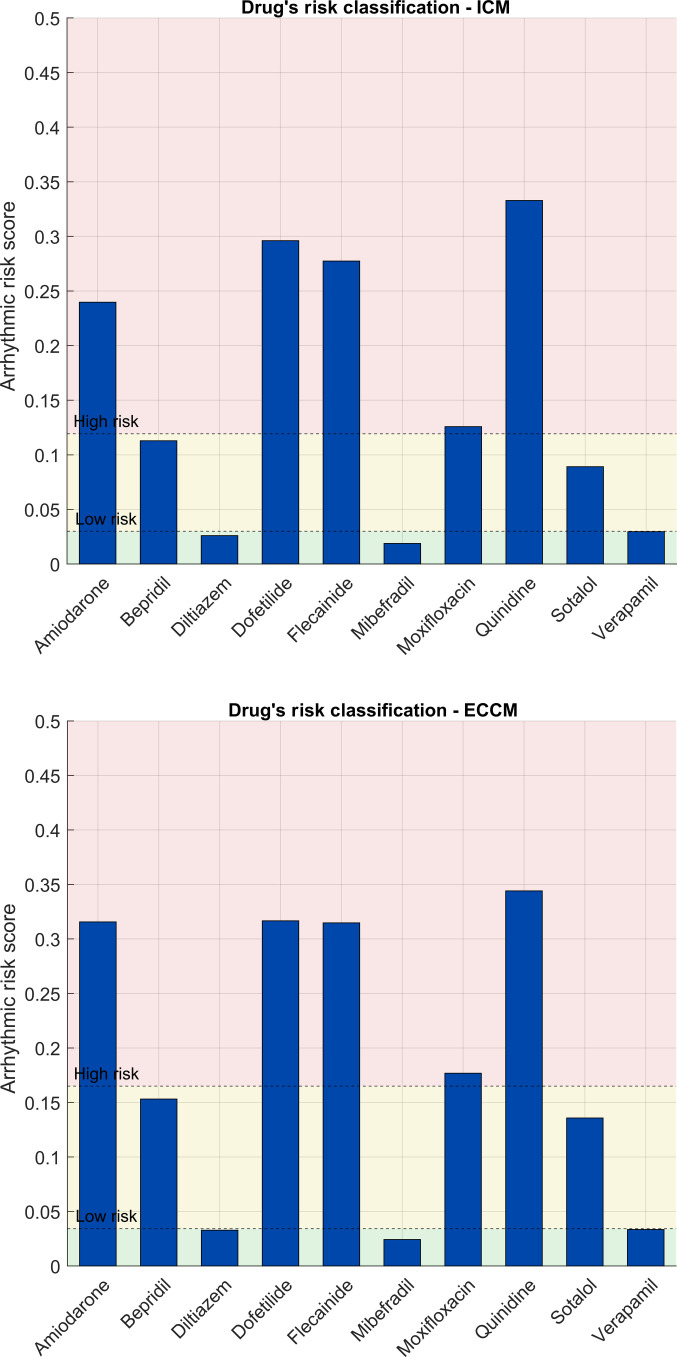
Comparison of arrhythmic risk scores for various drugs evaluated with 2 distinct models: the ICM (isolated-cell model) in the top panel and the ECCM (electrotonically coupled cell model) in the bottom panel. Each bar represents the risk score associated with a given drug. The pale green region highlights scores below the critical threshold (low risk), whereas the pale red region denotes scores above it (high risk). The dashed horizontal line indicates the threshold separating low from high risk. The low (ℓ) and high (*h*) thresholds were determined through the 2-dimensional grid-search procedure described in “Computation of the thresholds *l* and *h*” section, selecting the pair $\left(\ell^{*},\;h^{*}\right)$ that maximized the Macro-F1 score in the 3-class classification (low, borderline, and high). ICM thresholds: 0.029 to 0.116. ECCM thresholds: 0.034 to 0.165.

For the grouped bar plot, the same false-positive and false-negative counts are used but represented as adjacent horizontal bars, blue for false negatives and red for false positives, ordered according to the biomarker pair as follows:

For classification purposes, 2 thresholds are defined, $T_{\mathrm{low}}$ and $T_{\text{high}}$, separating the low-, borderline-, and high-risk classes. Dashed lines in Fig. 2 indicate the classification thresholds separating the low-, borderline-, and high-risk regions ($T_{\mathrm{low}}$ and $T_{\text{high}}$). These thresholds are derived from the 8-biomarker reference score and are used to assign classes when computing false positives and false negatives.

$$\begin{array}{c} \%{\mathrm{FP}}_{\left(i,j\right)}^{\left(k\right)}=100\cdot \frac{1}{N}\sum_{n}1_{\left\{{\hat{c}}_{n,\left(i,j\right)}=\text{High}\wedge c_{n}\neq \text{High}\right\}} \end{array}$$ (22A)

$$\begin{array}{c} \%{\mathrm{FN}}_{\left(i,j\right)}^{\left(k\right)}=100\cdot \frac{1}{N}\sum_{n}1_{\left\{{\hat{c}}_{n,\left(i,j\right)}\neq \text{High}\wedge c_{n}=\text{High}\right\}} \end{array}$$ (22B)

where $c_{n}\in \left\{\mathrm{Low},\;\text{Borderline},\;\text{High}\right\}$ denotes the true class obtained by applying the thresholds $T_{\mathrm{low}}$ and $T_{\text{high}}$ to the reference score $r_{d,t}^{\left(8\right)}$, while ${\hat{c}}_{n,\left(i,j\right)}$ represents the predicted class obtained from the 2-biomarker score $r_{d,t}^{\left(2\right)}$. The total percentage error is defined as ERR%=FP%+FN%. Finally, *k* indicates the class of interest: high (H), borderline (B), or low (L).

The results section primarily focuses on $\%{\mathrm{FP}}_{\left(i,j\right)}^{\left(H\right)}$ and $\%{\mathrm{FN}}_{\left(i,j\right)}^{\left(H\right)}$. This choice is motivated by evidence from medical and regulatory contexts, where avoiding the misclassification of high-risk cases is considered a priority. In particular, false negatives in the early stages of drug development can lead to substantial clinical and economic consequences [48]. In diagnostic pathology and pharmacovigilance, Council for International Organizations of Medical Sciences (CIOMS) VIII guidelines emphasize the importance of preventing the omission of critical safety signals, thereby identifying false negatives as a primary concern [49].

### Stimulation protocol and software

Both the ICM and ECCM were stimulated with a rectangular stimulus lasting 2 ms, set at twice the stimulation threshold. Subsequently, a train of 500 stimuli was delivered at a frequency corresponding to 75 beats per minute, with a basic cycle length (BCL) of 800 ms, reflecting a physiological sinus rhythm. Specifically, in the ECCM, the tissue preparation was stimulated at one end, allowing the signal to propagate in the direction along the fibers. The simulations were carried out using MATLAB software for the ICM and Elvira software [50] for the ECCM, with a time step of 0.02 ms.

## Results

### Comparison between ICM and tissue model

The novel aspect introduced by the ECCM is the electrotonic coupling, aimed at providing a more physiologically realistic description of cardiac electrophysiology. By enabling mutual interaction among cardiomyocytes following electrical stimulation, the electrical pulses propagating along myocardial fibers become more likely to generate stable action potentials, resembling the behavior observed within the functional syncytium where cells electrically interact. This electrotonic interaction contributes to mitigating the occurrence of localized proarrhythmic events.

The ARS indices are reported in Fig. 2, providing a direct comparison between the results obtained from the 2 models.

The 2 panels in Fig. 2 depict the ARS for all analyzed drugs, with the ICM model shown in the top panel and the ECCM model in the bottom panel. Overall, both models produce comparable ARS values and lead to identical drug classifications, despite some numerical differences.

More generally, the risk estimated using ICM tends to be slightly lower than that obtained with ECCM, although this difference is not statistically significant (Wilcoxon signed-rank test, P=0.617).

Our models and CredibleMeds consistently classify amiodarone as proarrhythmic, while Zhou et al. categorize it as borderline. This is notable as the drug remains commercially available. We classified bepridil as a borderline drug, which differs from other sources that label it as proarrhythmic. Sotalol was designated as a borderline-risk drug by our models and Zhou et al., although CredibleMeds classifies it as proarrhythmic. Furthermore, qNET studies showed a split classification for sotalol between high risk and borderline risk. All other drug classifications were in agreement across all studies and described in Table 3.

**Table 3.** Proarrhythmic risk assessment of different drugs has been reported. The first 2 columns correspond to our models, while the third column represents the isolated-cell model from Ref. [15]. The fourth column includes results from Refs. [46,47,58]; the fifth column corresponds to the CredibleMeds assessment and the last column refers to drugs that are currently on the market, or have been withdrawn. If no country is mentioned, the drugs are available in both the United States and the European Union. All the data from clinics have been collected from EMA/FDA archives.

Drugs	ICM	ECCM	Zhou et al.	qNET	CM	Clinics
Amiodarone	HR	HR	BR	–	HR	Used
Bepridil	BR	BR	HR	HR	HR	Not Used
Diltiazem	LR	LR	LR	LR	LR	Used
Dofetilide	HR	HR	HR	HR	HR	Used USA
Flecainide	HR	HR	HR	–	HR	Used
Mibefradil	LR	LR	–	–	–	Not Used
Moxifloxacin	HR	HR	HR	–	HR	Used
Quinidine	HR	HR	HR	HR	HR	Not Used
Sotalol	BR	BR	BR	HR/BR	HR	Used
Verapamil	LR	LR	LR	LR	–	Used

EMA, European Medicines Agency; FDA, Food and Drug Administration; ICM, isolated-cell model; ECCM, electrotonically coupled cell model; HR, high risk; BR, borderline risk; LR, low risk

### Classification performance

#### Class-wise F1 scores

To provide a more detailed evaluation of the classification performance, the F1 score was computed separately for each risk class using a one-versus-rest formulation.

Both the ICM and the ECCM produced the same confusion matrix in the 3-class classification task. Consequently, identical class-wise F1 scores were obtained for the 2 models: $\begin{array}{c} F1_{\text{nonPro}}=1.000,\;F1_{\text{borderline}}=0.667,\;F1_{\mathrm{pro}}=0.909. \end{array}$

The lower value observed for the borderline class reflects a single misclassification in which one proarrhythmic compound was assigned to the borderline category. No non-proarrhythmic compounds were incorrectly classified as proarrhythmic.

Overall, the resulting Macro-F1 score was 0.859 with a global accuracy of 90%.

#### Sensitivity analysis excluding the borderline class

To evaluate the robustness of the proposed methodology under class imbalance, an additional sensitivity analysis was performed by collapsing the 3 risk categories into a binary classification problem (proarrhythmic vs. non-proarrhythmic). In this setting, compounds labeled as intermediate and high risk were grouped into a single proarrhythmic class, while low-risk compounds were assigned to the non-proarrhythmic class.

When the models were fitted on the full dataset, both the ICM and the ECCM achieved perfect separation between the 2 classes (AUC = 1.00), yielding the following confusion matrix: $\begin{array}{c} \mathrm{TN}=3,\;\mathrm{FP}=0,\;\mathrm{FN}=0,\;\mathrm{TP}=7 \end{array}$

TN=3,FP=0,FN=0,TP=7 (23)

However, LOOCV highlights the limitations associated with the small dataset. In this configuration, the models were trained on only 9 compounds per fold, resulting in greater variability of the estimated coefficients.

Under LOOCV, the ICM produced an AUC of 0.571 with the following confusion matrix (threshold = 0.5): $\begin{array}{c} \mathrm{TN}=3,\;\mathrm{FP}=0, \end{array}$$\begin{array}{c} \mathrm{FN}=5,\;\mathrm{TP}=2 \end{array}$

TN=3,FP=0,FN=5,TP=2 (24)

In contrast, the electrotonically coupled model yielded a higher discriminative performance with an AUC of 0.857 and the following confusion matrix: $\begin{array}{c} \mathrm{TN}=3,\;\mathrm{FP}=0,\;\mathrm{FN}=1,\;\mathrm{TP}=6 \end{array}$

TN=3,FP=0,FN=1,TP=6 (25)

These results indicate that biomarkers derived from the electrotonically coupled formulation provide improved statistical separability between proarrhythmic and non-proarrhythmic compounds in the binary setting.

This analysis is reported only as a robustness test. The primary objective of the proposed methodology remains the 3-class risk stratification framework, which reflects the regulatory distinction between low, intermediate, and high proarrhythmic risk categories.

Within this original 3-class setting, both the ICM and the ECCM achieved identical performance, with a Macro-F1 score of 0.859 and an overall classification accuracy of 90%. LOOCV confirmed the stability of these results (LOO Macro-F1 = 0.859; accuracy = 90%).

#### Correlation analysis among biomarkers

To evaluate potential redundancy among biomarkers, we analyzed the correlation structure among electrophysiological descriptors using a pairwise Spearman correlation analysis. The analysis revealed moderate to high correlations between several biomarkers, particularly those associated with repolarization abnormalities such as APD prolongation and triangulation.

Such correlations are expected from a physiological perspective, as several electrophysiological biomarkers reflect different manifestations of reduced repolarization reserve and repolarization instability [51–53]. Consequently, partial dependencies among these descriptors do not necessarily imply methodological redundancy but rather reflect shared underlying electrophysiological mechanisms.

Interestingly, the correlation structure differs between isolated-cell simulations (ICM) and electrotonically coupled tissue simulations (ECCM). In the tissue model, several biomarkers associated with cellular-scale instabilities appear attenuated compared to the isolated-cell setting. This behavior is consistent with the stabilizing effect of electrotonic coupling, whereby local electrophysiological instabilities may be partially suppressed due to the source–sink balance in electrically coupled cardiac tissue [54–56].

As a consequence, repolarization-related descriptors, particularly APD90 and triangulation, emerge as the most prominent electrophysiological drivers in the ECCM simulations. However, as shown in the manuscript, restricting the classification to these descriptors alone leads to an increase in false-positive predictions. Further insights are reported in the Supplementary Materials.

### Sample size and biomarker weight effects on the arrhythmic risk index

In the previous paragraphs, we introduced 8 distinct biomarkers to comprehensively characterize the ARS that has been reported in Fig. 1 and we used a sufficiently large number of cells to adequately describe the pro-arrhythmic behavior of drugs, in accordance with [57]. Conversely, in this section, we assess the combined impact of cellular subsampling (100 vs. 2,989 cells) and biomarker reduction (from 8 to 2) on the final arrhythmic risk index using 2 complementary visual representations.

#### Sensitivity analysis of the number of resampling iterations

To evaluate the number of Monte Carlo resampling iterations required to obtain stable estimates of the ARS, a sensitivity analysis was performed. Random subsets of 100 cells were extracted from the full simulated population (*n* = 2,989), and the ARS was recomputed for each resampling iteration. The procedure was repeated for $N=\left\{10,50,100,200,400\right\}$ iterations. For each value of *N*, the variance of the estimator of the mean ARS was computed. The resulting estimator variances were $9.09\times 10^{-7}$,$1.43\times 10^{-7}$,$1.03\times 10^{-7}$,$5.50\times 10^{-8}$, and $2.36\times 10^{-8}$ for N=10, 50, 100, 200, and 400, respectively. These values correspond to standard errors of approximately $9.54\times 10^{-4}$, $3.78\times 10^{-4}$, $3.21\times 10^{-4}$, $2.35\times 10^{-4}$, and $1.54\times 10^{-4}$. For reference, the ARS values observed across drugs range approximately from 0.03 to 0.38. The uncertainty associated with the estimator obtained using *N* = 200 iterations therefore represents less than 1% of the smallest ARS value in the dataset. Increasing the number of iterations beyond 200 produces only marginal reductions in estimator variance while increasing computational cost proportionally. Based on this analysis, 200 resampling iterations were considered sufficient to obtain numerically stable ARS estimates.

#### Analytical relationship between subsample size and ARS variance

To investigate the effect of subsample size on the stability of the ARS, the variance of the score was computed for increasing numbers of randomly sampled cells extracted from the full simulated population (*n* = 2,989). The analysis was conducted independently for the ICM and the ECCM. For each subsample size, multiple random subsets were generated and the variance of the resulting ARS values was computed. The resulting variance trends are reported in the Supplementary Materials. To quantify the relationship between subsample size and ARS variance, a regression analysis was performed in log–log space using the model. Thus, the observed variance trends are consistent with the theoretical behavior expected from Monte Carlo sampling, whereby increasing the number of sampled cells reduces the variability of the estimated quantity due to the progressive reduction of sampling noise. Overall, this analysis applied to both ICM and ECM configurations demonstrates that increasing the number of sampled cells progressively stabilizes ARS estimation, while the presence of electrotonic coupling further attenuates variability at the tissue level. Detailed regression statistics are provided in the Supplementary Materials.

#### Bubble plots

In the bubble plots, each 2-biomarker pair is compared against the 8-biomarker baseline by calculating, for each pair and each drug, the median bias, defined as the difference between the median of the 200 risk scores obtained with 2 biomarkers and that obtained with the 8-biomarker model. This quantity is evaluated as a function of the RMSE computed over the same distribution of 200 subsampling iterations with samples of 100 cells.

**Fig. 3.**
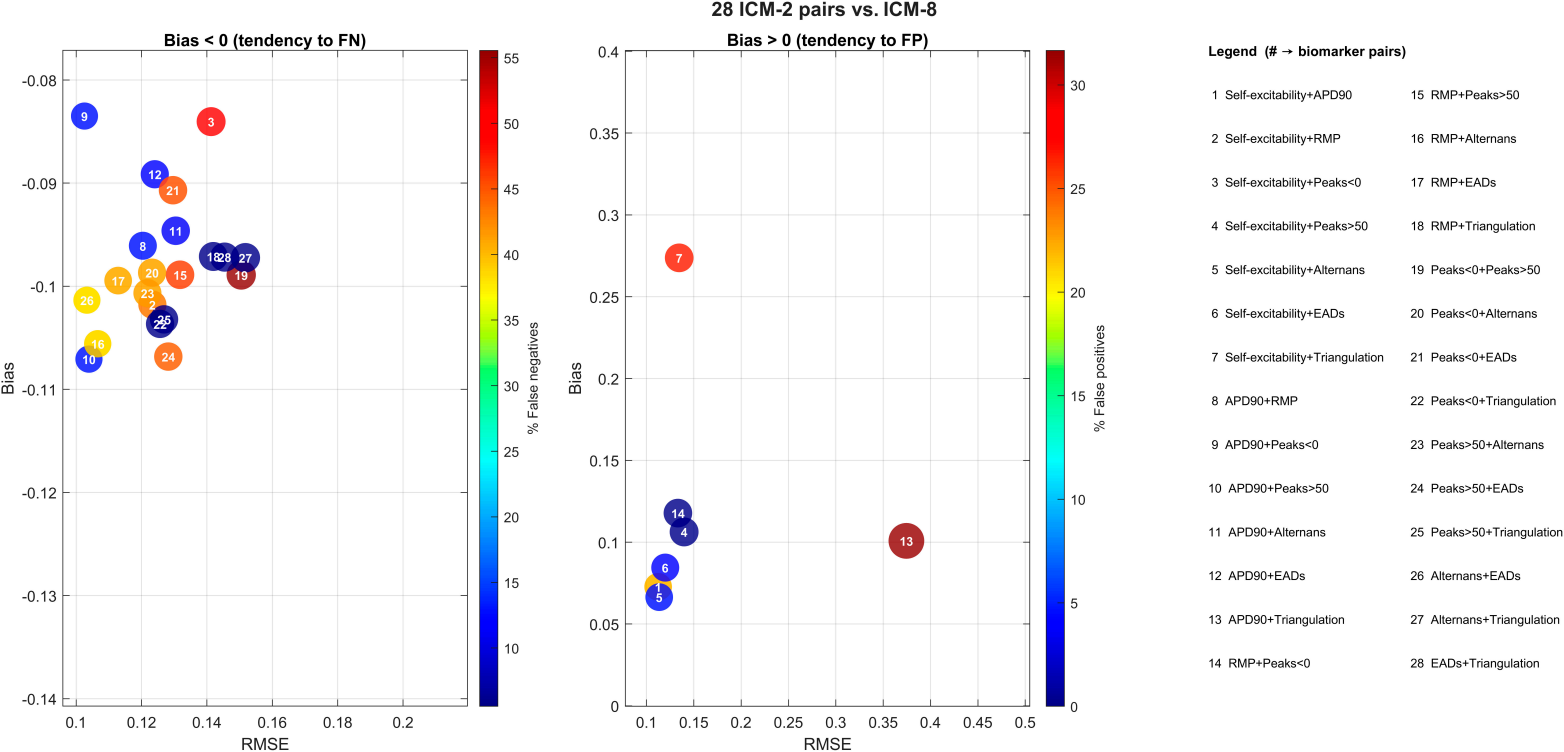
Comparison between risk scores obtained with 2-biomarker pairs (ICM-2) and the 8-biomarker reference score (ICM-8). In the left panel, negative bias (tendency to false negatives) is shown: the *x*-axis reports the mean RMSE over 200 iterations, and the *y*-axis reports the mean bias (${\text{median}}_{\mathrm{ICM}-2}$ − ${\text{median}}_{\mathrm{ICM}-8}$) within [−0.14, −0.08]. Bubble sizes are proportional to the √RMSE rule and the color scale indicates the percentage of cells classified as nonarrhythmic by the 2-biomarker model but arrhythmic by the 8-biomarker model (false negatives). In the right panel, positive bias (tendency to false positives) is shown with the *y*-axis in [0, 0.4]; color indicates the percentage of false positives and bubble sizes follow the √RMSE rule.

In the left panel of Fig. 3, all biomarker pairs exhibit a negative median bias, indicating a tendency to underestimate risk (i.e., classifying an excessive number of pro-arrhythmogenic events as non-pro). Most bubbles cluster around a bias of approximately −0.10 and an RMSE between 0.10 and 0.15, revealing a systematic underestimation. The dark-blue coloration of many bubbles corresponds to false-negative rates up to approximately 15%, whereas only a few pairs in this panel exceed this incidence (warmer colors).

In the right panel, only a limited number of pairs display a positive bias, indicating a tendency to overestimate risk (false positives). Notably, pair 7 (Self-excitability + Triangulation) stands out with bias≈0.27, RMSE≈0.13, and more than 30% false positives (large red bubble), whereas the remaining pairs remain below 10% (blue bubbles).

The pairs located closest to the origin, characterized by low RMSE≈0.10 and bias values near zero (−0.08 to −0.10), best reproduce the behavior of the ICM-8 model. For example, pairs 1 (Self-excitability + APD90), 5 (Self-excitability + Alternans), and 14 (esting membrane potential [RMP] + Peaks<0) exhibit minimal overall error together with low rates of both false negatives and false positives. Nevertheless, the reduction from 8 to 2 biomarkers introduces a systematic shift toward underestimation, as evidenced by the clustering of bubbles in the left panel.

It is important to note that, although some bias and RMSE values approach zero, these deviations must be interpreted relative to the observed score range (approximately 0.03 to 0.31). A deviation of 0.10 therefore represents roughly one-third of the full range and should be considered a practically substantial error.

**Fig. 4.**
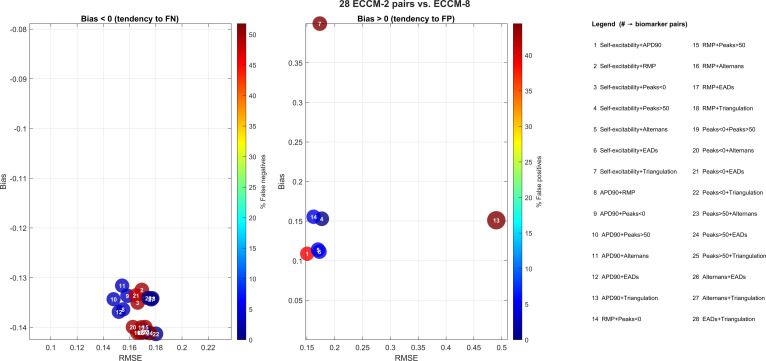
Comparison between risk scores obtained with 2-biomarker pairs (ECCM-2) and the 8-biomarker reference score (ECCM-8). In the left panel, negative bias (tendency to false negatives) is shown: the *x*-axis reports the mean RMSE over 200 iterations, and the *y*-axis reports the mean bias (${\text{median}}_{\text{ECCM}-2}$ − ${\text{median}}_{\text{ECCM}-8}$) within [−0.14, −0.08]. Bubble sizes are proportional to the √RMSE rule and the color scale indicates the percentage of cells classified as nonarrhythmic by the 2-biomarker model but arrhythmic by the 8-biomarker model (false negatives). In the right panel, positive bias (tendency to false positives) is shown with the *y*-axis in [0, 0.4]; color indicates the percentage of false positives and bubble sizes follow the same √RMSE rule. Furthermore, the pairs at the bottom that are squeezed in the plot are referring to 15 to 17, 19, 20, and 22 to 26.

Furthermore, in the left panel of Fig. 4 (bias <0, tendency toward false negatives), nearly all biomarker pairs underestimate risk, clustering around a median bias of approximately −0.135 and an RMSE between 0.15 and 0.18. The deep-blue shading indicates false-negative rates up to approximately 10%, with only a few pairs approaching about 15% (lighter colors).

In the right panel (bias >0, tendency toward false positives), only 3 biomarker pairs overestimate risk. Pair 7 (Self-excitability + Triangulation) again dominates, with bias≈0.40, RMSE≈0.15, and more than 45% false positives (dark red). The remaining overestimating pairs (e.g., pair 13) remain below approximately 15% false positives.

Similar conclusions regarding relative deviations can be drawn for both ECCM and ICM configurations.

Overall, when a reduced subset of biomarkers is combined with small cellular samples, the comparative analysis of the results presented in Figs. 3 and 4 highlights 2 main observations. First, regarding false positives (right panels), the ICM and ECCM models show substantial agreement, capturing nearly identical behaviors and patterns. However, a notable difference emerges in the analysis of false negatives (left panels): the ECCM model exhibits a stronger tendency to produce false negatives compared with the ICM model.

#### Stacked-bar plots

**Fig. 5.**
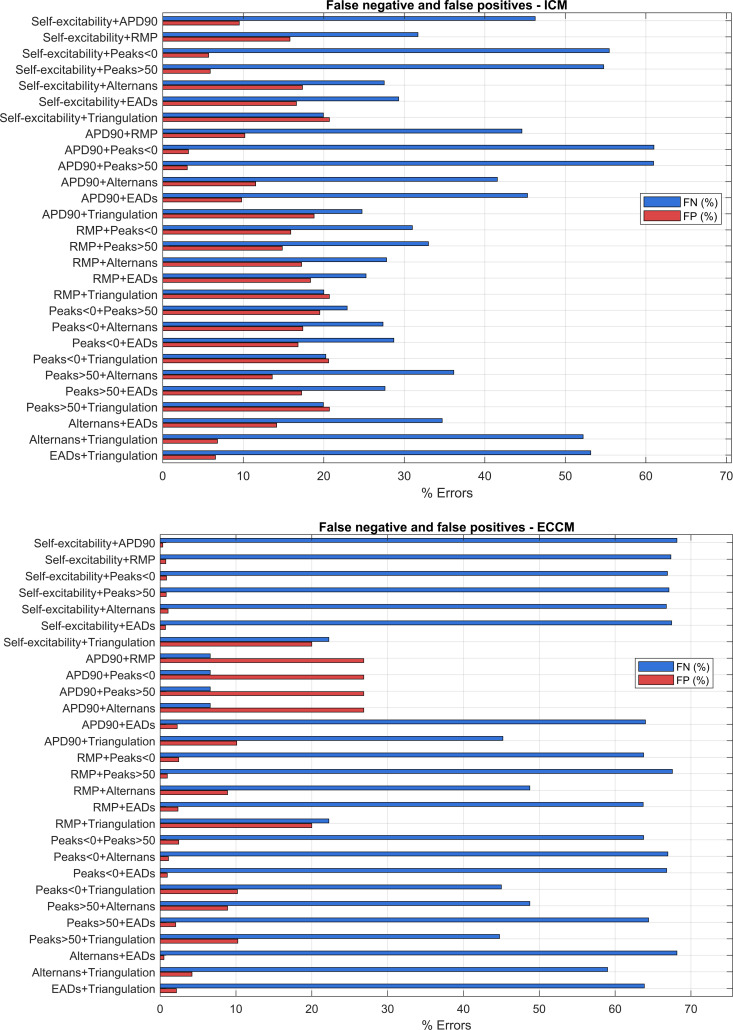
In the top panel, the ICM model is depicted, while the ECCM is shown in the bottom panel. For each of the 28 pairs (*i*,*j*) of biomarkers, horizontal bars of false negatives (FN in blue) and false positives (FP in red) expressed in percentage over the totality of the observations (100 subcells for 200 iterations) are shown on the *y*-axis. For each observation *n*, the reference score is the ICM-8 score and the ICM-2 score is defined in detail in Methods, as well as the %FP and %FN

In Fig. 5 each row represents a biomarker pair $\left(i,\;j\right)$. The blue bar %FN measures how many times the pair misses a case that the 8-biomarker model classifies as high (underestimation of risk), while the red bar %FP measures how many times the pair overestimates a case that the 8-biomarker model does not consider high. The overall length (blue + red) corresponds to the total error relative to the reference. In the ECCM panel, %FN are systematically high in almost all pairs (often 40% to 60%). This indicates that, in the coupled tissue simulations, restricting the analysis to only 2 biomarkers tends to underestimate the arrhythmic risk compared with the complete ARS index. This effect reflects the loss of electrophysiological information caused by the reduction of predictors.

In the ICM panel (isolated cell), the error remains high but shows greater variability among pairs: some combinations accumulate more FP (overestimation), while others accumulate more FN (underestimation). In any case, the total error remains consistent, confirming that 2 biomarkers do not capture the complexity of the risk profile synthesized by the 8-biomarker model.

Comparing the 2 panels, when downgrading to the 2-biomarker model, the error associated with ECCM is more severe and the missing information weighs more heavily and leads to missed reports of high-risk evaluations.

#### Comparison with the FDA qNET metric

To provide a reference with respect to the CiPA framework, we also evaluated the qNET metric using the ECCM simulations, for which the ionic currents required for charge integration were available. For each drug, qNET values were computed at the cellular level and aggregated using the median across the simulated population.

The resulting drug classification was largely consistent with the ARS-based framework. In particular, most compounds retained the same risk category. An interesting difference was observed for bepridil, which was classified as borderline by the ARS index but as non-proarrhythmic according to qNET. This observation suggests that the 2 approaches capture similar electrophysiological information, although they rely on different mechanistic descriptors (action-potential morphology versus net ionic charge balance). Additional details are provided in the Supplementary Materials.

In conclusion, the reduction from 8 to 2 biomarkers consistently introduces a negative bias in the great majority of pairs across both models. This translates to an underestimation of risk and an increase in false negatives, confirming that a substantial amount of information is lost when using only 2 biomarkers in both the ICM and ECCM contexts.

This suggests that electrotonic coupling modifies which combinations lead to risk overestimation without altering the global trend. The error remains primarily FN-dominated, and a 2-biomarker approximation is consistently unreliable. The most performant pairs (low RMSE, near-zero bias) are similar between models, but even these exhibit a nonnegligible deviation given the score range of 0 to 0.31. The conclusion is therefore consistent across both models: reducing to 2 biomarkers is not a reliable approach. The cross-model consistency of this finding underscores its robustness and generalizability.

### Statistical power considerations

The present proof-of-concept study was conducted on a limited dataset (*n* = 10 drugs; class counts $n_{\mathrm{low}}=4$, $n_{\text{border}}=1$, and $n_{\text{high}}=5$). Consequently, statistical power is inherently constrained and performance estimates may exhibit sensitivity to individual compounds.

LOOCV was therefore performed to evaluate the robustness of the classification results. Under this protocol, the model achieved a Macro-F1 score of 0.859 with an overall accuracy of 90%.

Bootstrap resampling was additionally used to evaluate the stability of the global risk-score thresholds. The resulting confidence intervals were relatively narrow for the low-risk boundary (ICM: 0.0268 to 0.0363; ECCM: 0.0299 to 0.0512), whereas the threshold separating borderline- and high-risk compounds exhibited wider variability (ICM: 0.0846 to 0.2763; ECCM: 0.1201 to 0.3145).

Finally, the borderline class is represented by a single compound in the present dataset; therefore, class-specific metrics for this category should be interpreted cautiously.

## Discussion

The present study introduces a proof-of-concept framework for the standardized assessment of drug-induced proarrhythmic risk based on the integration of multiple electrophysiological biomarkers derived from in silico simulations. The proposed ARS combines 8 descriptors of action potential morphology into a composite index that summarizes the overall electrophysiological instability induced by a compound.

The primary objective of this work is methodological rather than predictive. Specifically, the study aims to introduce and evaluate a standardized framework for aggregating electrophysiological biomarkers into a composite ARS and to assess its behavior under different modeling conditions. Despite the intentionally limited pharmacological dataset, the results demonstrate that the proposed approach produces consistent classifications across both isolated-cell simulations (ICM) and electrotonically coupled tissue simulations (ECCM), achieving an overall accuracy of approximately 90% with a Macro-F1 score of 0.859 under LOOCV.

### Physiological interpretation of biomarker contributions

The ARS framework relies on the integration of multiple electrophysiological descriptors capturing complementary manifestations of repolarization instability. Rather than relying on a single biomarker, the composite score aggregates information from several mechanisms known to be associated with proarrhythmic activity, including APD prolongation, repolarization morphology alterations, and cellular instability phenomena.

This multiparametric formulation offers 2 main advantages. First, it preserves the interpretability of individual biomarkers while combining their contributions into a single quantitative index. Second, it mitigates the fragility of simple rule-based approaches that classify compounds as proarrhythmic when a single biomarker crosses a predefined threshold. In the presence of biological variability and correlations among electrophysiological descriptors, such binary rules tend to amplify noise and increase false positive rates. In contrast, the weighted aggregation adopted in ARS allows robust electrophysiological signals to dominate the final classification.

Interestingly, the relative contribution of biomarkers differs between isolated-cell and electrotonically coupled simulations. In isolated-cell conditions, several instability-related descriptors contribute to the final score, including APD90 prolongation, early afterdepolarizations, triangulation, abnormal action potential peak amplitudes (reflecting insufficient or excessive depolarization), and alternans-related indicators. When electrotonic coupling is introduced, however, the contribution becomes largely dominated by repolarization descriptors such as APD90 prolongation and triangulation. This behavior is physiologically consistent with the stabilizing effect of electrotonic coupling, which tends to suppress localized cellular instabilities while preserving tissue-level manifestations of delayed repolarization [52,55,56].

### Impact of biomarker reduction and cellular subsampling on risk classification

An additional aspect emerging from the present analysis concerns the combined impact of reducing the number of biomarkers and subsampling the cellular population used to estimate the ARS. While simplified frameworks based on a limited number of descriptors may appear attractive due to their conceptual simplicity, our results indicate that both biomarker reduction and reduced cellular sampling can substantially affect the stability of the risk estimation.

When the full 8-biomarker ARS is approximated using only 2 biomarkers, a systematic deviation from the reference score is observed. In most cases this deviation manifests as a negative bias, corresponding to an underestimation of the arrhythmic risk and therefore to an increased probability of false-negative classifications. Only a limited number of biomarker pairs exhibit the opposite behavior, producing positive bias and a higher rate of false positives.

This behavior can be interpreted in light of the multivariate nature of drug-induced electrophysiological perturbations. Proarrhythmic mechanisms typically affect several aspects of action potential morphology simultaneously, including repolarization duration, repolarization dynamics, and instability-related phenomena. Restricting the analysis to a small subset of descriptors therefore removes part of the electrophysiological information contained in the full biomarker set.

At the same time, the use of reduced cellular populations introduces an additional source of variability in the estimated risk score, reflecting the intrinsic heterogeneity of the simulated cell population. Together, these effects highlight the importance of both multiparametric biomarker integration and sufficiently large cellular populations for obtaining stable and reliable estimates of drug-induced proarrhythmic risk.

Consequently, simplified models based on very few biomarkers or limited cellular samples may fail to capture relevant electrophysiological signatures and may therefore increase the probability of both false-negative and false-positive classifications. These findings support the rationale for adopting multiparametric frameworks in computational drug-induced pro-arrhythmia risk assessment, where integrating complementary biomarkers and adequately representing cellular variability can provide a more robust characterization of drug-induced electrophysiological instability.

### Role of electrotonic coupling

Electrotonic coupling represents an essential physiological feature of cardiac tissue, as cardiomyocytes are electrically connected through gap junctions and operate within a functional syncytium. The resulting source–sink interactions smooth local voltage gradients and dampen isolated cellular instabilities that may arise in single-cell simulations.

In the present study, the ECCM produced pharmacological classifications identical to those obtained with the ICM, while ARS values remained statistically comparable between models (Wilcoxon signed-rank test, P=0.617). Rather than indicating redundancy, this observation suggests that the arrhythmogenic signal captured by the biomarker set reflects pharmacologically driven mechanisms that persist even under electrotonic interactions.

Therefore, the introduction of ECCM does not aim to improve predictive performance per se, but rather to verify that the proposed biomarker aggregation approach remains stable when cell-to-cell electrical coupling is introduced.

### Comparison with existing computational frameworks

Several computational frameworks have been proposed to assess drug-induced proarrhythmic risk, including the CiPA initiative and the qNET metric derived from ionic charge integration. In contrast to these approaches, the ARS index is based on descriptors of action potential morphology and electrophysiological instability rather than on net ionic charge balance.

To provide a methodological reference, we evaluated the qNET metric using the ECCM simulations, for which the required ionic current recordings were available. The resulting classifications were largely consistent with those obtained using the ARS framework, with most compounds retaining the same risk category. An interesting difference was observed for bepridil, which was classified as borderline by ARS and as non-proarrhythmic according to qNET. Bepridil is known to exhibit complex multichannel pharmacology, simultaneously blocking several inward and outward currents, including $I_{\mathrm{Kr}}$ and $I_{\mathrm{CaL}}$. The balance between these opposing electrophysiological effects has been shown to produce variable predictions in in silico frameworks depending on the model configuration and drug concentration [14,34,47]. Such behavior may partly explain why both ARS and qNET deviate from the expected proarrhythmic classification in the present simulations.

Overall, this agreement indicates that, despite relying on different electrophysiological descriptors, the 2 metrics converge toward similar risk assessments.

Moreover, some differences between the classifications obtained in this study and those reported in previous investigations may also arise from methodological differences in biomarker selection. For example, some studies relied primarily on a limited set of biomarkers, including RMP and early after-depolarizations (EADs). As shown in the present analysis, restricting the evaluation to a reduced biomarker subset increases the probability of false-negative classifications, highlighting the importance of multiparametric frameworks capable of capturing a broader range of electrophysiological perturbations [15].

### Interpretation of drug-specific discrepancies

The contribution of individual biomarkers to the final ARS classification should be interpreted within the multivariate structure of the model. Because the score is computed as a weighted combination of biomarker probabilities, the resulting classification reflects the overall electrophysiological profile induced by each compound rather than the behavior of a single descriptor.

This aspect may partly explain the discrepancies observed for some drugs when comparing the present results with previous computational or clinical classifications. For instance, amiodarone has been classified as borderline risk in some computational studies based on limited biomarker sets, whereas broader multiparametric frameworks may capture additional electrophysiological signatures [14,15,34].

Similarly, the proarrhythmic profile of sotalol has shown context-dependent behavior in several modeling studies, reflecting the selective nature of its $I_{\mathrm{Kr}}$ block and the dependence of computational predictions on the biomarkers considered [14,34].

Regarding bepridil, as previously discussed, the drug was classified as borderline risk in our simulations and its ARS value lies close to the high-risk threshold. In addition, only limited proarrhythmic events were observed at therapeutic concentrations. Variability in pharmacokinetic exposure, which is not explicitly represented in the present simulations, could plausibly influence this classification.

Differences in effective plasma concentration across individuals may therefore shift the electrophysiological response toward higher-risk profiles.

### Study limitations and statistical considerations

The main limitation of the present study is the intentionally small pharmacological dataset used to demonstrate the methodological framework. The analysis includes 10 compounds distributed across 3 risk classes, with a single borderline instance. Consequently, statistical power is limited and performance metrics may exhibit sensitivity to individual drugs.

To partially address this limitation, several complementary validation strategies were implemented. LOOCV was used to evaluate the robustness of the classification results, yielding a Macro-F1 score of 0.859 with an overall accuracy of 90%. Bootstrap resampling was additionally employed to assess the stability of the classification thresholds. The resulting confidence intervals were relatively narrow for the low-risk boundary (ICM: 0.0268 to 0.0363; ECCM: 0.0299 to 0.0512), whereas the threshold separating borderline- and high-risk compounds exhibited wider intervals. This behavior is expected given the limited number of compounds and the presence of a single borderline drug in the dataset.

Importantly, the 8-biomarker ARS used in the biomarker reduction analysis should be interpreted as an internal reference model rather than as an external clinical ground truth. The purpose of this analysis was methodological and to quantify the information loss associated with the reduction of the number of biomarkers, rather than to establish absolute predictive superiority.

### Future perspectives

The present work represents the first step of a broader research program aimed at developing standardized computational methodologies for proarrhythmic risk assessment. An extended analysis including approximately seventy pharmacological compounds is currently underway. The larger dataset will allow a more comprehensive evaluation of biomarker weighting strategies, threshold stability, and predictive performance across a wider pharmacological spectrum.

Future developments will also investigate the integration of population-based modeling strategies and additional electrophysiological descriptors in order to further refine the characterization of drug-induced repolarization abnormalities.

Overall, the results presented here suggest that multiparametric frameworks integrating multiple electrophysiological biomarkers may provide a robust and physiologically interpretable strategy for the computational assessment of drug-induced proarrhythmic risk.

## Data Availability

All data supporting the findings of this study are available within the article and its Supplementary Materials. No additional restrictions apply.
