# Supplementary material for: Toward Standardized Methodologies for Drug-Induced Proarrhythmia Classification: An In Silico Proof of Concept
Source: Comput Struct Biotechnol J. 2026 Apr 10;35(1):0041. doi: 10.34133/csbj.0041 (PMC13067877; doi:10.34133/csbj.0041)
Supplement: Supplementary 1 — Figs. S1 to S5 [file csbj.0041.f1.docx]

**SUPPLEMENTARY MATERIALS**

Matteo Costi, Jose Maria Ferrero and Jose Felix Rodriguez Matas

LaBS - CompBiomech, Dept. of Chemistry, Materials and Chemical engineering "G. Natta", Politecnico of Milan, 20133, Milan, Italy.

Center for Research and Innovation in Bioengineering (Ci2B), Universitat Politècnica de València - 46022, Valencia, Spain

**Geometrical structure of the tissue patch**

**
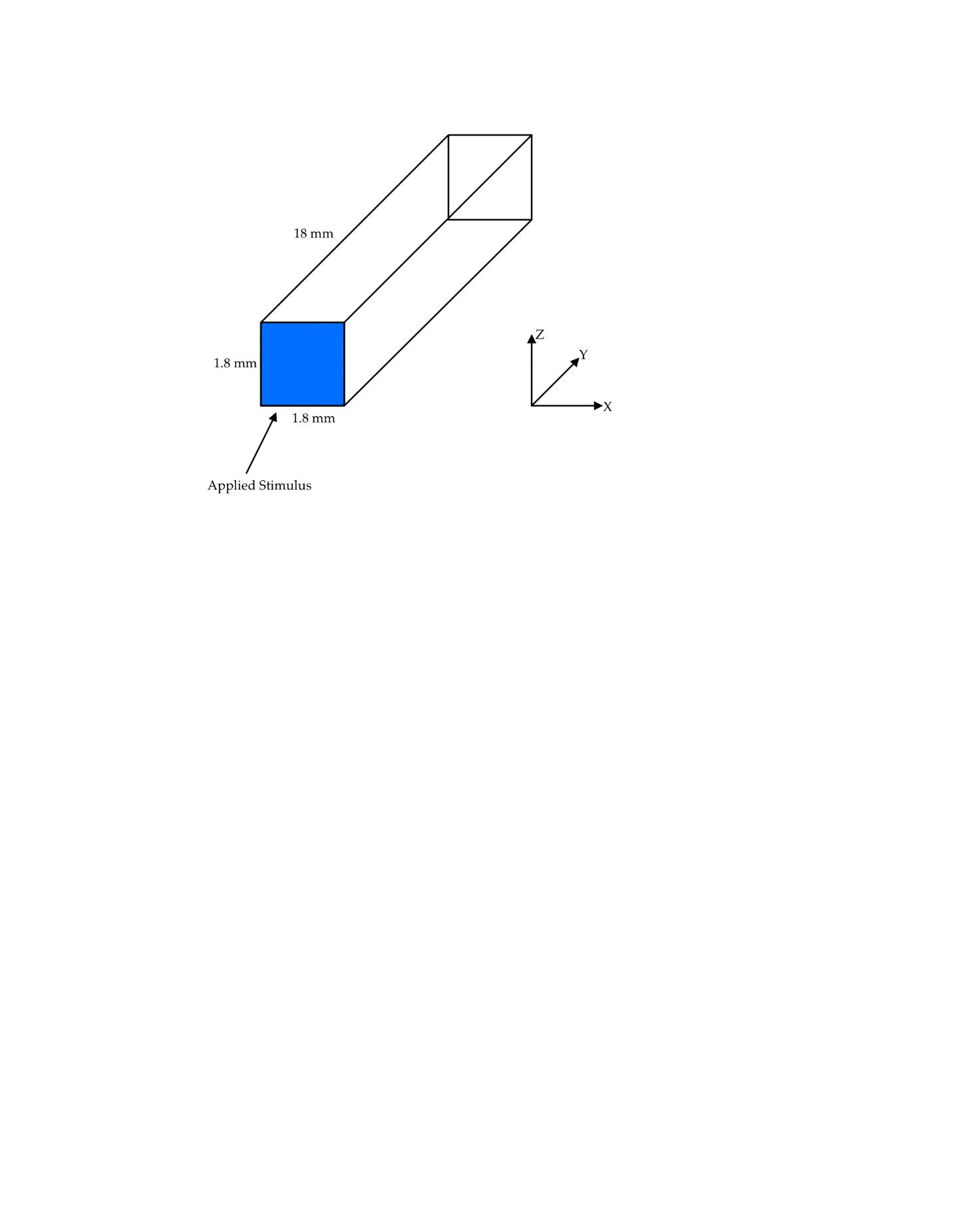
**

Figure 1 – Representation of the parallelepiped used to model the tissue preparation. The blue face represents the surface of stimulation. The fibers are oriented along the longitudinal direction (y-axis).

**Detailed biomarkers analysis**

All the biomarkers used in our analysis were chosen to encompass an heterogenous range of instabilities in action potential morphology, reason why we have decided to adopt the label "Arrhythmic Risk Score" (ARS) instead of "TdP Risk Score". This adjustment aims to provide a broader perspective on arrhythmia risk beyond TdP alone. These biomarkers were implemented to detect variations indicative of potential arrhythmic events and aiming to ensure accuracy in our assessment.

In this work, biomarkers $B1, \ldots, Bk$ are implemented as binary indicator variables (event detectors).

For each action potential, the underlying electrophysiological quantity associated with Bk (e.g., APD prolongation, resting potential, alternans, EADs) is computed and compared against a literature-based threshold. We then set Bk=1 if the criterion is met (pro-arrhythmic event detected), and Bk=0 otherwise.

This procedure defines an algorithmic filter that identifies action potentials exhibiting instability features. Whenever a biomarker detects a pro-arrhythmic event, it contributes to the estimation of the probability of the corresponding arrhythmic outcome under drug exposure. This approach not only allows for a comprehensive evaluation of action potential instabilities but also provides insight into the likelihood of the occurrence of an arrhythmic event in response to pharmacological intervention.

B1 is defined as the maximum time derivative of the membrane potential during the resting phase, computed as:

$$D_{rest}=\max_{t\in rest} \left( \frac{dV_{m}}{dt} \right)$$

Where “rest” indicates the resting phase.

This real-valued quantity is subsequently compared against a threshold (0.01 mV/ms) to detect abnormal spontaneous depolarization. The binary indicator is defined as:

$$B1=\left\{ \begin{aligned} 1, &if D_{rest}> 0.01 \\ 0, &otherwise \end{aligned} \right.$$

B2 (APD90 prolongation) quantifies relative APD90 prolongation with respect to control conditions.

Let ${APD}_{90}^{Drug}$ and ${APD}_{90}^{Control}$ denote the APD90 measured in drug and control conditions, respectively (evaluated on the last paced beat).

The relative prolongation is computed as:

$$\Delta_{APD90}\frac{{APD}_{90}^{Drug}-{APD}_{90}^{Control}}{{APD}_{90}^{Control}}$$

Accordingly, B1 is defined as:

$$B2=\left\{ \begin{aligned} 1, &if \Delta_{APD90}> 0.2 \\ 0, &otherwise \end{aligned} \right.$$

This criterion identifies excessive repolarization prolongation exceeding 20% relative to baseline. This value is not intended to represent a clinically validated arrhythmogenic threshold. Rather, based on pharmacological safety evidence, a $\geq$ 20% prolongation of APD90 was considered an operational cut-off to identify marked repolarization delay, which is commonly regarded as biologically relevant in preclinical electrophysiology studies [1,2].

Biomarker B3 (resting membrane potential) evaluates alterations of the resting state. Let RMP denote the resting membrane potential measured at the beginning of the analyzed beat.

$$B3=\left\{ \begin{aligned} 1, &if RMP> -50mV \\ 0, &otherwise \end{aligned} \right.$$

This criterion detects abnormal depolarization of the resting membrane potential, indicative of impaired excitability.

To characterize B4 and B5 it is important to define the action potential peak for each paced beat, such that:

$$V_{peak}=\max_{t\in T_{beat}} V_{m}\left( t \right)$$

Where $V_{m}\left( t \right)$ is the membrane potential and $T_{beat}$ denotes the time interval corresponding to a single paced beat.

Two mutually exclusive biomarkers were defined as follows:

$$B4=\left\{ \begin{aligned} 1, &if V\_peak <-0 mV \\ 0, &otherwise \end{aligned} \right.$$

$$B5=\left\{ \begin{aligned} 1, &if V\_peak >50 mV \\ 0, &otherwise \end{aligned} \right.$$

Peaks within the physiological interval $0\leq V_{peak}\leq50 mV$ were considered normal, yielding B4=0 and B5=0. Therefore, B4 and B5 were implemented separately because they represent two qualitatively different electrophysiological instabilities (insufficient depolarization vs. excessive overshoot), rather than a single symmetric amplitude constraint. They are mutually exclusive by definition and were treated independently within the composite risk index.

Biomarker B6 (alternans) was computed by quantifying beat-to-beat relative variations in APD$_{90}$ over the last three paced beats.

Let $APD_{90}^{(n)}$denote the APD90 measured at beat “n”, with n=1,2,3 corresponding to the final three beats of the stimulation protocol. A tolerance tol=0.05 (5%) was adopted. Alternans are detected if at least one consecutive beat pair satisfies:

$$\frac{\left| APD_{90}^{(n)}-APD_{90}^{(n-1)} \right|}{APD_{90}^{(n)}}>0.05, n=2,3$$

Accordingly, B6 is defined as the binary indicator variable:

$$B4=\left\{ \begin{aligned} 1, &if the above condition holds for n=2 or n=3 \\ 0, &otherwise \end{aligned} \right.$$

This criterion captures dynamic repolarization instability by identifying relative beat-to-beat oscillations exceeding 5% under steady pacing conditions.

Biomarker B7 (EADs) was automatically computed using a slope-based criterion applied to the repolarization phase. An EAD is identified if there exists at least one time index k such that:

$$150 ms <t\left( k \right)<650 ms and diffV\left( k \right) >0.01 mV/ms$$

where diffV(k) represents the discrete temporal derivative of the membrane potential, computed between two consecutive time samples. Accordingly, the biomarker B7 is defined as:

$$B7=\left\{ \begin{aligned} 1, &if an EAD is detected \\ 0, &otherwise \end{aligned} \right.$$

For B8, the general idea underlying this biomarker was to first compute the triangulation in the control population and in the same population following drug administration. Triangulation was defined as the difference between APD90 and APD30. The relative (percentage) variation in triangulation induced by the drug was then calculated with respect to the control condition. This variation was subsequently compared to a predefined threshold value. If the percentage increase in triangulation exceeded 20%, the binary biomarker was set to 1; otherwise, it was set to 0.

Triangulation was computed as:

$$TRI=APD90-APD30$$

The relative variation in triangulation was defined as:

$$\Delta TRI=\frac{TRI_{Drug}-TRI_{Control}}{TRI_{Control}}$$

The biomarker B8 was defined as:

$$B8=\left\{ \begin{aligned} 1, &\Delta TRI\geq0.2 \\ 0, &otherwise \end{aligned} \right.$$

Triangulation of the action potential is a well-recognized marker of repolarization morphology alteration and pro-arrhythmic liability. In pharmacological and in silico investigations, relative increases in triangulation of approximately 20% (and greater) have been explicitly reported in association with drug-induced repolarization abnormalities [3,4]. Although no universally accepted threshold exists, a $\geq$ 20% relative increase represents a substantial deviation from baseline morphology and was therefore adopted here as an operational criterion to flag marked triangulation enhancement.


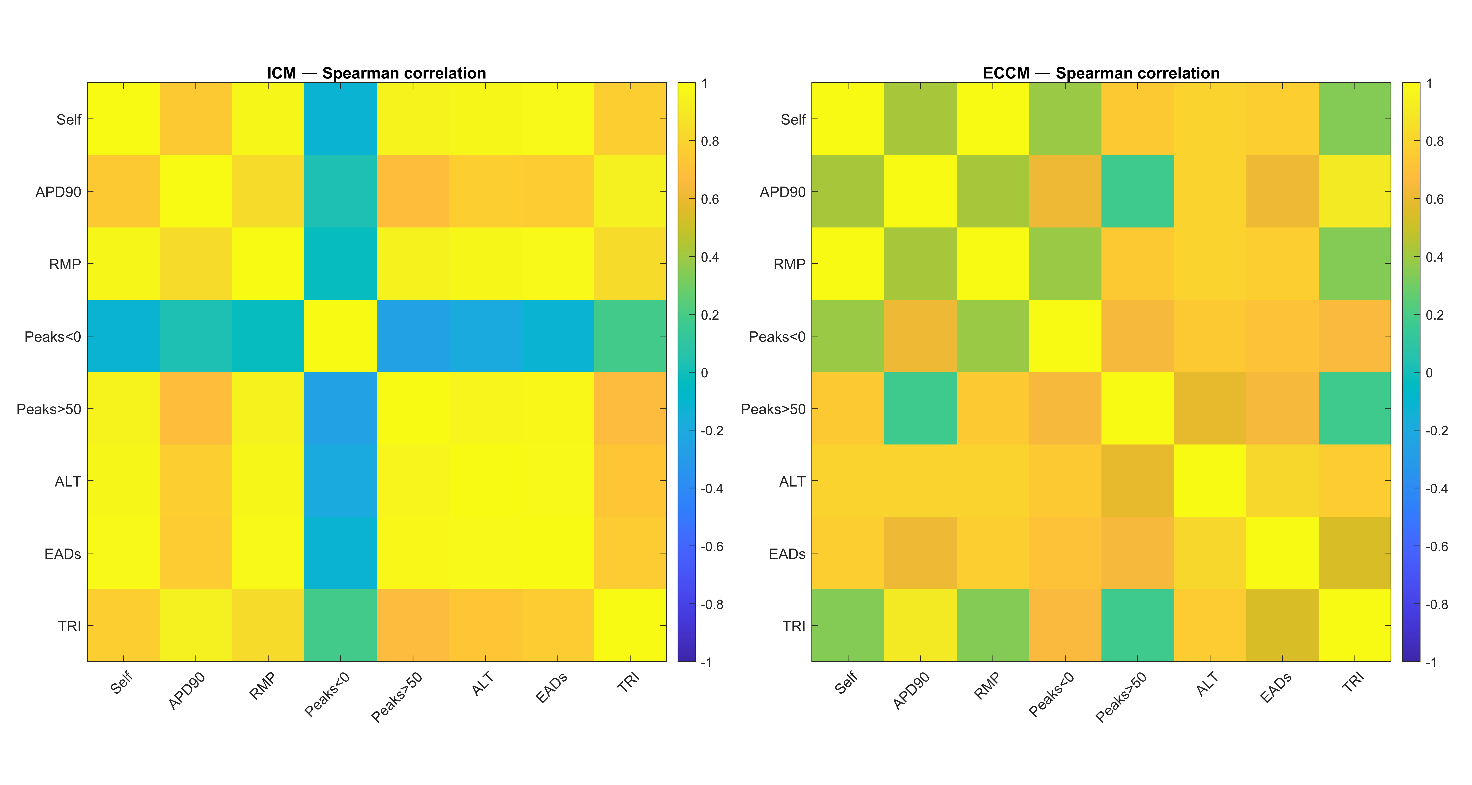
**Pairwise Spearman correlation among electrophysiological biomarkers**

Figure 2 - Spearman correlation matrices computed across all analysed compounds for isolated-cell simulations (ICM, left) and electrotonically coupled tissue simulations (ECCM, right). Colours represent the rank correlation coefficient $\rho$ between each pair of biomarkers.

Pairwise Spearman correlation matrices computed among all electrophysiological biomarkers for

isolated-cell simulations (ICM, left) and electrotonically coupled tissue simulations (ECCM, right).

Each matrix reports the rank-based correlation coefficient ($\rho$) between pairs of biomarkers

computed across the analyzed compounds.

Moderate to high correlations are observed among several repolarization-related descriptors,

particularly APD90, triangulation (TRI), and other markers associated with repolarization dynamics.

Such dependencies are expected because multiple electrophysiological biomarkers reflect different

manifestations of reduced repolarization reserve and repolarization instability.

Compared with the isolated-cell simulations, several correlations appear attenuated in the ECCM

configuration. This behaviour is consistent with the stabilizing effect of electrotonic coupling,

whereby local electrophysiological instabilities may be partially damped by the source–sink balance

in electrically coupled cardiac tissue.

**Subsample size and ARS variance scaling**

**
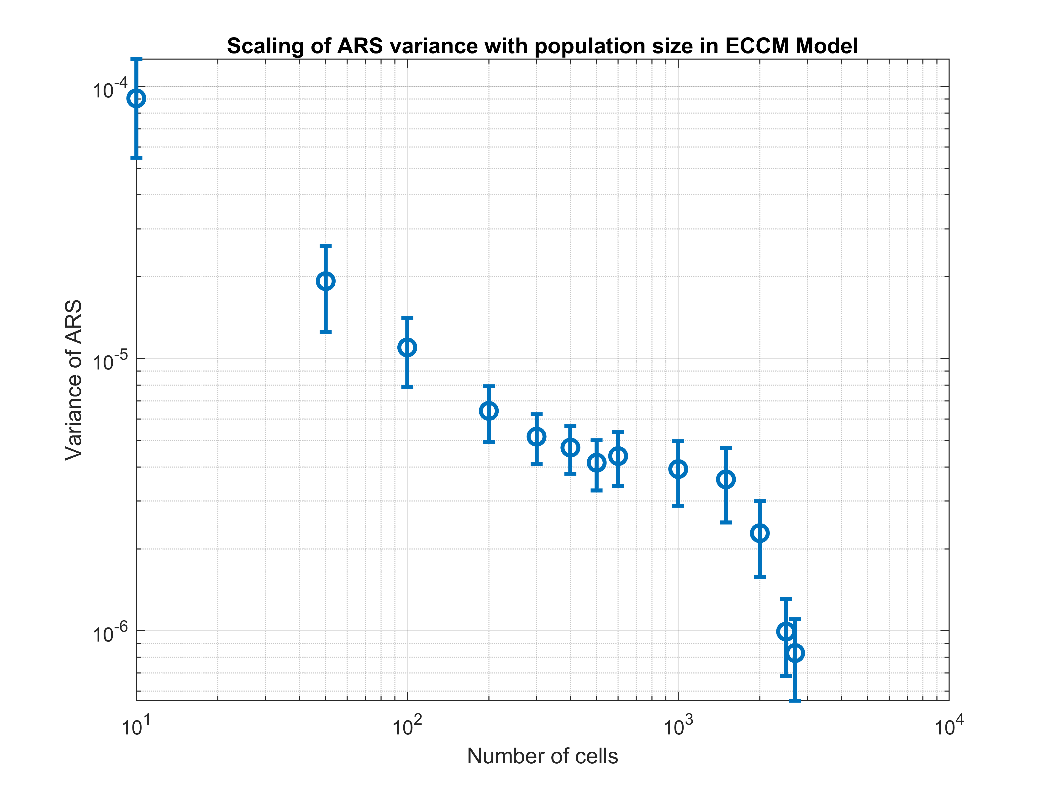
**

Figure 4 -Scaling of the variance of the Arrhythmic Risk Score (ARS) as a function of the number of sampled cells in the electrotonically coupled cell model (ECCM). Each point represents the variance of the ARS obtained from repeated random subsampling of the full cellular population (n=2989). Error bars denote the standard deviation across subsampling realizations. Both axes are shown in logarithmic scale. The decreasing trend indicates that increasing the number of sampled cells progressively stabilizes the estimated ARS in the presence of electrotonic coupling.

**
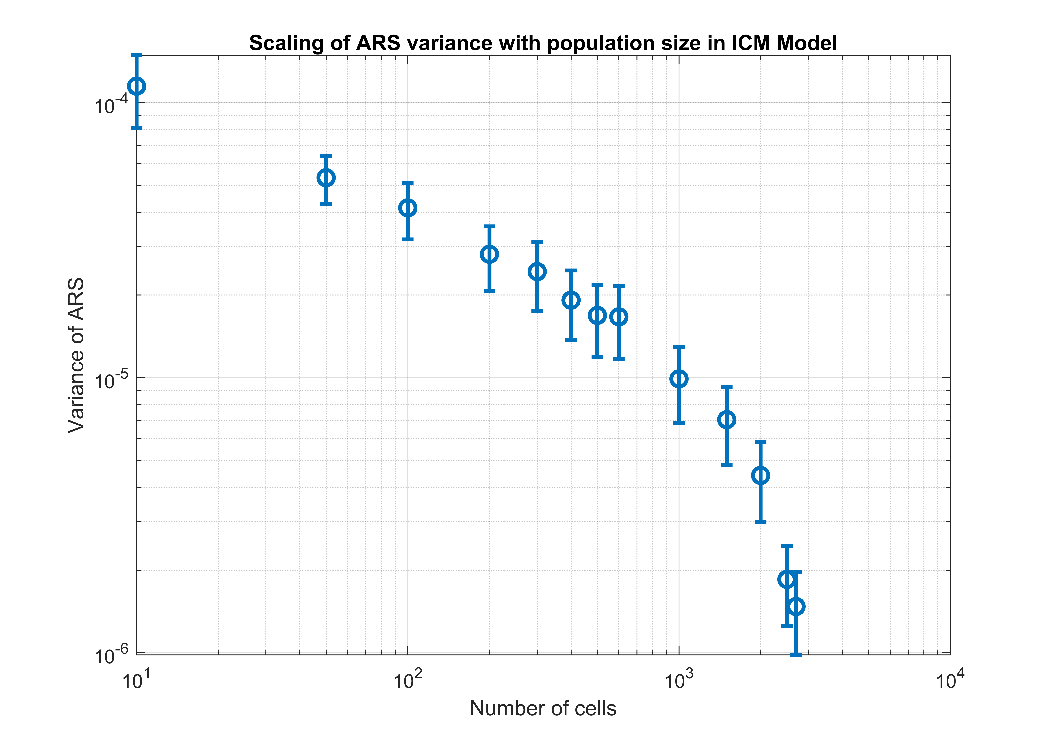
**

Figure 3 - Scaling of the variance of the Arrhythmic Risk Score (ARS) as a function of the number of sampled cells in the isolated cell model (ICM). Each point represents the variance of the ARS computed from repeated random subsampling of the full cellular population (n=2989). Error bars indicate the standard deviation across subsampling realizations. Both axes are shown in logarithmic scale. The results show a systematic decrease of ARS variance with increasing population size, consistent with the expected variance reduction of Monte Carlo estimators.

To evaluate how the variability of the Arrhythmic Risk Score (ARS) depends on the number of sampled cells, a sensitivity analysis was performed using increasing subsample sizes extracted from the full cellular population (n=2989). For each subsample size N, multiple random subsets of cells were generated and the ARS was recomputed. The variance of the resulting ARS values was then calculated.

This analysis was conducted independently for the isolated cell model (ICM) and the electrotonically coupled cell model (ECCM). The resulting variance trends are shown in Figure3 and Figure4

To quantitatively characterize the relationship between subsample size and ARS variance, the data were modeled in log–log space using the following regression model:

$$\log\left( var(ARS) \right)=\alpha+\beta log(N)$$

where N denotes the number of sampled cells.

For the ICM configuration, the regression produced: $\alpha=-6.877$, $\beta= -0.8436$ and $p < {10}^{-5}$

For the ECCM configuration, the regression produced: $\alpha=-8.433$, $\beta= -0.7525$ and $p < {10}^{-5}$

In both cases, the negative value of $\beta$ indicates a systematic decrease of ARS variance as the number of sampled cells increases. The exponent values close to -1 are consistent with the theoretical behavior expected for Monte Carlo estimators, where the variance of the estimator decreases approximately proportionally to 1/N.

Notably, the slope obtained for the ECCM configuration is slightly less steep than for the isolated-cell model, indicating that electrotonic coupling reduces the sensitivity of the ARS to cellular subsampling.

This behavior is consistent with the stabilizing effect of electrical coupling in cardiac tissue, which tends to smooth local electrophysiological variability across neighboring cells.

**Threshold stability analysis**

Bootstrap resampling was used to estimate the confidence intervals of the classification thresholds. The resulting intervals for the *low-risk* boundary were relatively narrow:

Low Risk: (ICM: 0.0268–0.0363; ECCM: 0.0299–0.0512)

Indicating a stable separation between low-risk and potentially pro–arrhythmic compounds.

In contrast, the threshold separating borderline and *high-risk* drugs exhibited wider intervals:

High Risk: (ICM: 0.0846–0.2763; ECCM: 0.1201–0.3145)

Reflecting the limited sample size and the presence of a single borderline compound in the dataset.

**Comparison with the qNET metric**


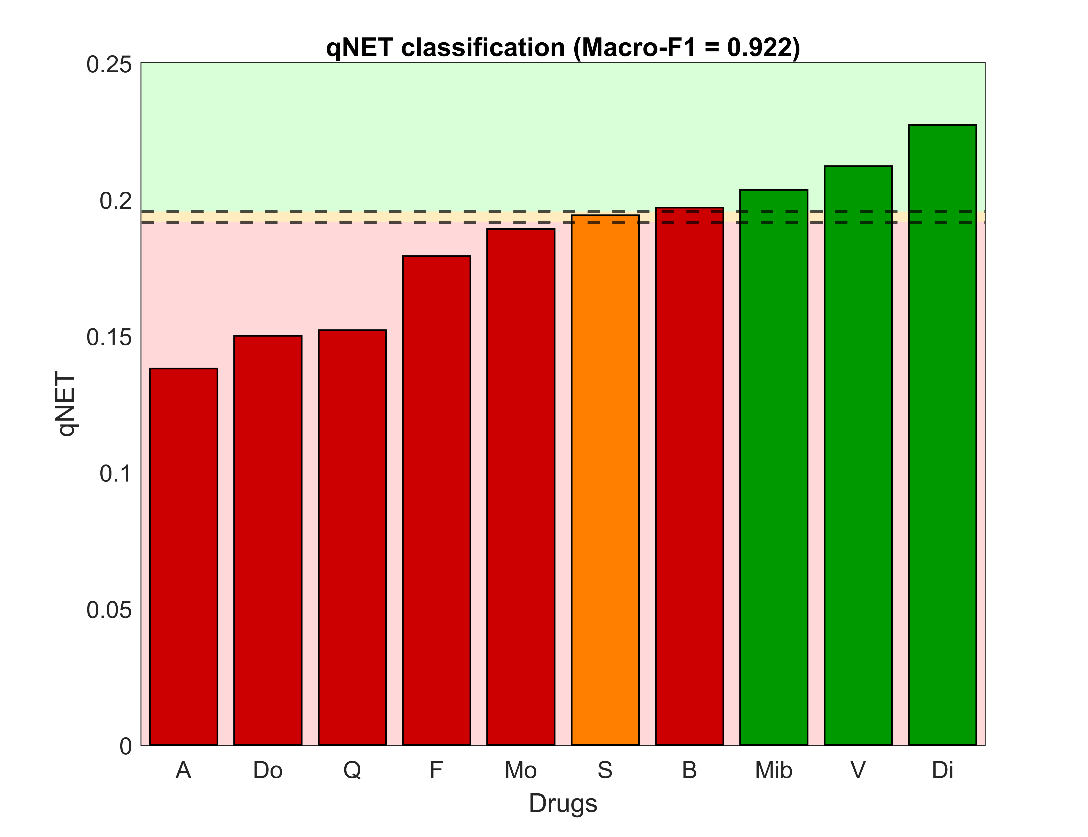


Figure 5- Drug classification based on the qNET metric computed from ECCM simulations - Cellular qNET values were aggregated using the median across the simulated population, and classification thresholds were optimized using the same Macro-F1 procedure adopted for the ARS framework.
Red indicates pro-arrhythmic risk, green non pro-arrhythmic, and orange borderline.

As an additional reference with respect to the CiPA framework, we evaluated the qNET metric using the electrotonically coupled cellular model (ECCM) simulations. Because ionic currents were stored during ECCM simulations, cellular qNET values could be directly computed and subsequently aggregated at the drug level using the median across the simulated tissue population. To ensure a fair comparison with the Arrhythmic Risk Score (ARS), the qNET-based classification was obtained using the same statistical pipeline adopted for ARS, including the optimization of classification thresholds through Macro-F1 maximization.

The resulting classification is reported in Figure5. The ordering of compounds is largely consistent with that obtained using the ARS framework, with most drugs retaining the same qualitative risk stratification. This agreement suggests that both approaches capture comparable pro-arrhythmic trends, despite relying on different electrophysiological descriptors. In particular, while qNET is based on the integration of ionic currents, the ARS framework relies on action potential morphology biomarkers, yet leads to a similar overall classification. A notable difference is observed for Bepridil, which lies close to the classification boundary and is classified as non–pro-arrhythmic according to qNET, whereas it was identified as borderline using ARS.

**REFERENCES**

[1] S. Redfern, L. Carlsson, A. S. Davis, W. G. Lynch, I. Mackenzie, S. Palethorpe, P. K. S. Siegl, I. Strang, A. T. Sullivan, R. M. Wallis, A. J. Camm, and T. G. Hammond. Relationships between preclinical cardiac electrophysiology, clinical qt interval prolongation and torsade de pointes for a broad range of drugs: evidence for a provisional safety margin in drug development. Cardiovascular Research, 58(1):32–45, 2003.

[2] Bernard Fermini, Jules C. Hancox, Najah Abi-Gerges, Matthew Bridgland-Taylor, Kamal Chaudhary, Thomas Colatsky, Kim Correll, William Crumb, Bryan Damiano, et al. A new perspective in the field of cardiac safety testing through the comprehensive in vitro proarrhythmia assay paradigm. Journal of Pharmacological and Toxicological Methods, 81:9–14, 2016.

[3] Qu, H. M. Vargas, and D. G. Strauss. Action potential recording and pro-arrhythmia risk assessment: Relationships between apd90, triangulation and beat-to-beat instability. Frontiers in Physiology, 8:1109, 2018.

[4] B. Trenor et al. In silico assessment of drug safety in human ventricular myocytes: modeling of repolarization abnormalities. PLoS ONE, 8(4):e61212, 2013.
